# Population impact of fine particulate matter on tuberculosis risk in China: a causal inference

**DOI:** 10.1186/s12889-023-16934-8

**Published:** 2023-11-18

**Authors:** Jun-Jie Mao, Hong-Lin Chen, Chun-Hu Li, Jia-Wang Lu, Yuan-Yuan Gu, Jian Feng, Bin Zhang, Jun-Feng Ma, Gang Qin

**Affiliations:** 1 Joint Division of Clinical Epidemiology, Affiliated Hospital of Nantong University, School of Public Health of Nantong University, Nantong, China; 2 Jiangyin Center for Disease Control and Prevention, Wuxi, China; 3 Department of Epidemiology and Biostatistics, School of Public Health of Nantong University, Nantong, China; 4 Department of Infectious Diseases, Affiliated Hospital of Nantong University, Nantong, China; 5 Centre for the Health Economy, Macquarie University, Sydney, NSW Australia; 6 National Key Clinical Construction Specialty - Department of Respiratory and Critical Care Medicine, Affiliated Hospital of Nantong University, Nantong, China; 7 Nantong Center for Disease Control and Prevention, Nantong, China

**Keywords:** PM_2.5_, Tuberculosis, Causality, Empirical dynamic modeling, Eco-driver

## Abstract

**Background:**

Previous studies have suggested the potential association between air pollution and tuberculosis incidence, but this association remains inconclusive and evidence to assess causality is particularly lacking. We aimed to draw causal inference between fine particulate matter less than 2.5 μm in diameter (PM_2.5_) and tuberculosis in China.

**Methods:**

Granger causality (GC) inference was performed within vector autoregressive models at levels and/or first-differences using annual national aggregated data during 1982–2019, annual provincial aggregated data during 1982–2019 and monthly provincial aggregated data during 2004–2018. Convergent cross-mapping (CCM) approach was used to determine the backbone nonlinear causal association based on the monthly provincial aggregated data during 2004–2018. Moreover, distributed lag nonlinear model (DLNM) was applied to quantify the causal effects.

**Results:**

GC tests identified PM_2.5_ driving tuberculosis dynamics at national and provincial levels in Granger sense. Empirical dynamic modeling provided the CCM causal intensity of PM_2.5_ effect on tuberculosis at provincial level and demonstrated that PM_2.5_ had a positive effect on tuberculosis incidence. Then, DLNM estimation demonstrated that the PM_2.5_ exposure driven tuberculosis risk was concentration- and time-dependent in a nonlinear manner. This result still held in the multi-pollutant model.

**Conclusions:**

Causal inference showed that PM_2.5_ exposure driving tuberculosis, which showing a concentration gradient change. Air pollutant control may have potential public health benefit of decreasing tuberculosis burden.

**Supplementary Information:**

The online version contains supplementary material available at 10.1186/s12889-023-16934-8.

## Background

Tuberculosis (TB) is a chronic infectious disease and one of the leading causes of mortality worldwide. *Mycobacterium tuberculosis* (*M. tb*) infects approximately one quarter of the world’s population (latent TB infection, LTBI) [1], causing estimated 10.0 million symptomatic cases and 1.4 million death in 2019 [2]. In addition, post-TB sequelae add substantially to the overall disease burden [3]. China accounted for 8.5% (rank 3rd, after India and Indonesia) of global total TB cases in 2019, and was included in WHO’s three high TB burden country lists for the period 2016–2020 [2].

The linkage between poverty and TB has long been apparent. In China, prenatal and early-life exposure to malnutrition during the Great Famine of 1959–1961 increased the risk of tuberculosis in adulthood [4]. On the one hand, China’s economic growth, accompanied by improved nutrition and better healthcare programs, has become an integral component of national TB control efforts [5]. On the other, rapid urbanization and large flow of migrant workers might facilitate TB transmission and spatial diffusion [6]. Furthermore, there is evidence suggesting an association between ambient air pollution (especially particulate matter 2.5, PM_2.5_), a byproduct of economic activity, and TB development [7–10]. However, it is methodologically complex to establish causal link between air pollution and TB because TB changes during the past four decades are unlikely to have happened without changes in other environmental and socio-economic conditions [11].

Developments in epidemiologic and statistical methods have brought light to better causal inference in disease ecology [12]. Standard regression-based methods suffer from both omitted variable bias and errors-in-variable bias. As our study subject is large, complex, coupled human-natural system, it is probable that the overall resilience of the system cannot be reduced to a linear relationship. Both Granger causality (GC) and convergent cross mapping (CCM) tests are powerful methodological approaches that can help distinguish causality from spurious correlation in time series from stochastic or deterministic (chaotic) dynamical systems [13].

There was a demonstrable affirmative correlation between ambient PM_2.5_ levels and the incidence of newly diagnosed pulmonary tuberculosis in Jinan, China [14]. However, the situation in Beijing was characterized by equivocal evidence, with no definitive positive link observed [15]. A recent finding on the causal impact between major PM_2.5_ components and TB showed that PM_2.5_ components exposure was associated with increased TB burden [16]. Studies examining the long-term effects of ambient air pollution on the incidence of TB remain sparse, particularly in the context of causal inference. In the study, we focused on PM_2.5_, using combined modeling analysis on a large dataset covering 31 provinces in mainland China, to explore the population impact of air pollution on TB at national and provincial scale.

## Methods

### Data

The longitudinal data was retrieved from provincial and national TB prevalence surveys [5, 17, 18]. The time series data of annual reported number of pulmonary tuberculosis (PTB) in China during 1982–2019 was collected from online global TB database (https://worldhealthorg.shinyapps.io/tb_profiles/) [19]. The panel data of TB incidence in 31 provinces (annually during 1997–2018, and monthly during 2004–2018), was obtained from Chinese public health science data center (https://www.phsciencedata.cn/).

The air pollutant and whether data were retrieved from the modern-era retrospective analysis for research and applications version 2 (MERRA-2) released by national aeronautics and space administration (NASA) of USA [20].

The national and provincial-level data on annual birth rate, population density, per capita GDP, certified doctors and beds of medical institutions were extracted from the governmental statistical yearbooks (http://www.stats.gov.cn/tjsj/ndsj/).

### Granger causality (GC) tests

GC tests are well-suited for rudimentary linear causality analysis, particularly in instances characterized by limited data length [21]. The GC analysis was conducted as an initial step to explore the causal relationship between PM_2.5_ and TB with the annual 1982–2019 time series data, using vector autoregressive (VAR) models [22] or vector error correction (VECM) models [23]. Then, the heterogeneous panel GC tests were applied to annual 1997–2018 and monthly 2004–2018 panel data, based on Monte-Carlo or Bootstrap simulation [24].

The analyses were performed using the standard modules (e.g., var, vec, vargranger, xtgcause) in Stata 17.0 (StataCorp, Texas, USA).

### Convergent cross mapping (CCM) method

The Granger causality framework is inapplicable in scenarios where the segregation of information pertaining to variables from the broader system is unfeasible, particularly in cases where causal relationships exhibit weak to moderate strengths. Conversely, CCM presents a heightened utility in addressing intricate systems and data, exhibiting diminished susceptibility to the effects of noise and external factors [25]. Nonetheless, it is imperative to note that CCM necessitates the availability of time-series data of substantial duration for meaningful analysis. Thus, we used empirical dynamic modeling (EDM), a data-driven equation-free mechanistic approach [25], to model mechanisms forcing TB epidemics with monthly 2004–2018 panel data. Convergent cross-mapping (CCM) method was adopted to distinguish causality between pairs of time series from correlations. The basic idea of CCM is to look for the signature of X in Y’s time series [26].

The convergent cross-mapping analysis, an EDM for detecting causality in nonlinear dynamic systems, [25] was composed of three parts here. First, the CCM causality between PM_2.5_ and TB incidence was tested based on univariate state-space reconstruction (SSR) according the modified methods described elsewhere [27, 28]. We examined whether the cross-map prediction skill (ρCCM, the Pearson correlation between observations and CCM prediction) increased and demonstrated convergence as the library length increased if causality existed for two variables. CCM for the real time series need to show higher prediction skill than 90% confidence intervals of surrogate time series.

Second, multivariate SSR (including stochastic causal variables as a coordinate in the state space) could improve the ability of nearest-neighbor prediction. For seasonal TB, PM_2.5_ could be considered stochastic because information about it may already be included in the univariate embedding [25]. We examined multivariate SSR forecast improvement, according to a modified method developed by a previous study [28].

Third, Scenario exploration with multivariate SSR was employed to investigate the effect of a small change in the potential driver (PM_2.5_) on TB incidence across different states of the system. The effect of ΔTB/ΔPM_2.5_ provided a way to understand the causality direction.

The analyses were performed using rEDM package version 0.7.5 of R software (R Foundation for Statistical Computing, Vienna, Austria).

### Distributed lag nonlinear model (DLNM)

While CCM helped us to establishing the causal relationship (statistical significance) and the causal direction (temporality), it provided little information on the causal strength (exposure-response relationship). Thus, we further evaluated the exposure risks using distributed lag nonlinear models (DLNM) [29].The basic model of DLNM is generalized linear model (GLM). In the multivariate DLNM, temperature, precipitation and sunshine duration were included to control the potential confounders [9]. The cumulative relative risks (RRs) were calculated for different extents of exposure to PM_2.5_ within lag 0–15 months, as well as for every 10 µg/m^3^ of PM_2.5_. The reference values of PM_2.5_ was set as 15 µg/m^3^ according to WHO’s air quality guidelines (https://www.who.int/publications/i/item/9789240034228). In order to fit the nonlinear and delayed effects, we constructed “cross-basis” (bidimensional) function and depicted the effects of predictors and lags simultaneously. Moreover, we computed a three-dimensional model of PM_2.5_, lag months and risk of TB incidence into a hexahedron.

Sensitivity analysis was conducted by fitting multi-pollutant models to identify the robustness of the results. To avoid multicollinearity problem, the pollutant would be excluded if the Pearson correlation coefficient ≥ 0.7 [29].

**Fig. 1:**
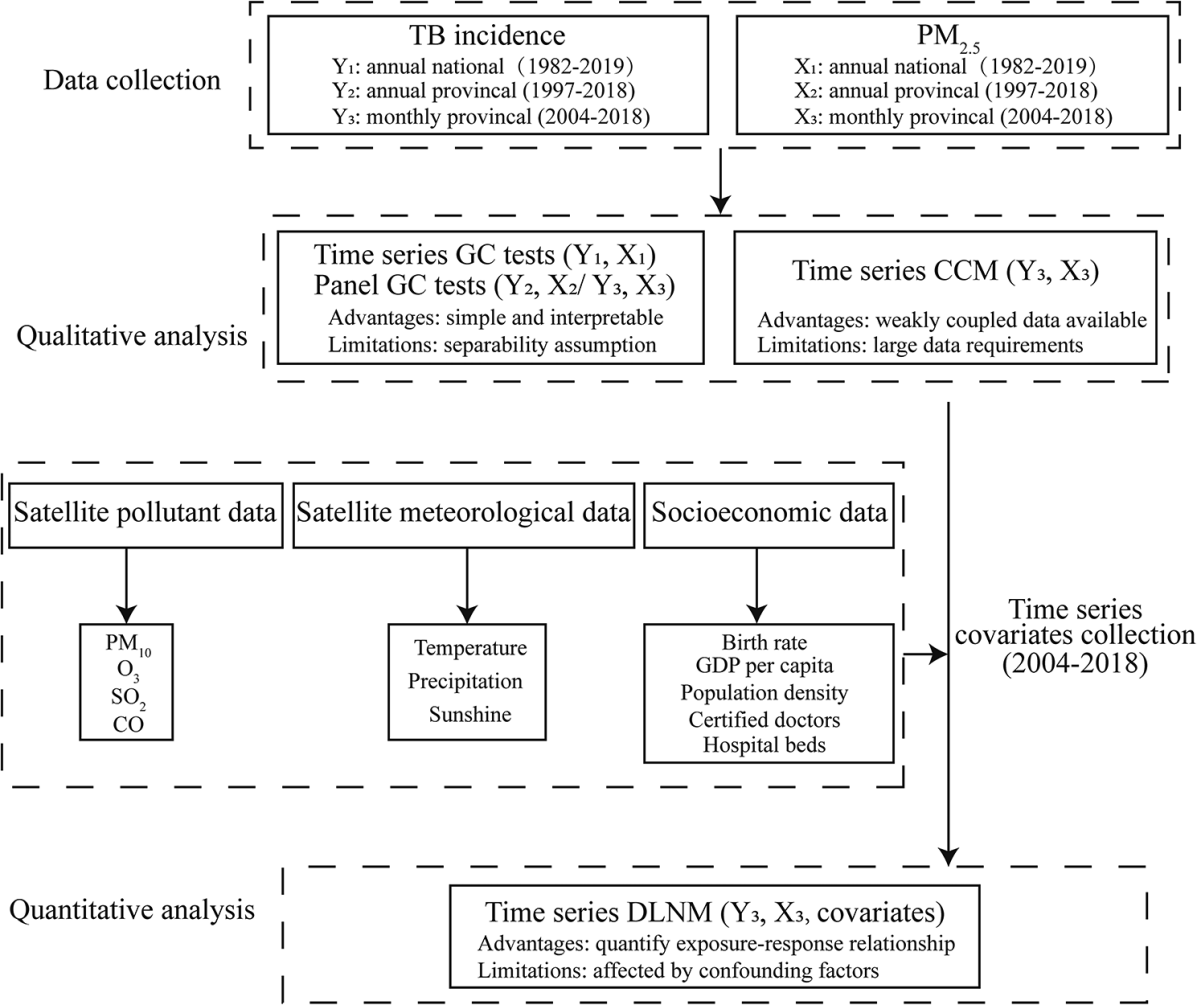
Methodology flowchart of the causal inference study on PM_2.5_ and TB.

The analyses were performed using the package “dlnm” version 2.4.7 in R software (R Foundation for Statistical Computing, Vienna, Austria). Figure 1 showed the complete flow diagram.

### Data availability

The data that supports the findings of this study are available in the supplementary material.

## Results

### Economic development and environmental health trends

With the progress of society, both PM_2.5_ and TB have experienced three stages during 1982–2019: from slow increase, then rapid rise to moderate decline (SI Appendix Fig. S1A, SI Appendix Table S1). Real GDP per capita (pGDP) is utilized to extend the environmental Kuznets curve (EKC) hypothesis to the interrelationships among economic growth, environment and health, indicated by the inverted U-shaped curves (SI Appendix Fig. S1B). That is, the health gains obtained through improved incomes could be significantly negated by the environmental stress variable at the beginning. But after a threshold of economic development level, environmental health issues will decline [30].

### GC analysis

We found positive associations between TB incidence and PM_2.5_ in most provinces during 1997–2018 (SI Appendix Fig. S2). Based on the VAR models using the non-stationary time series at difference, GC tests revealed a significant unidirectional causality from dPM_2.5_ to dTB (Wald F test, *P* = 0.026, Table 1, SI Appendix Table S2). The response of dTB to dPM_2.5_ reached its peak at 1-year and prevailed between 2 and 4 years (SI Appendix Fig. S3). Meanwhile, the GC analysis based on VECM also indicated a possible causal link from PM_2.5_ to TB, although the association did not reach statistical significance (*P* = 0.114) (Table 1).

**Table 1:** Granger causality between PM_2.5_ and tuberculosis

Test	Null hypothesis (H_0_)	Lag	Statistic	*P*	Conclusion
1982–2019 annual country-level data					
VAR-based GC	ΔPM_2.5_ does not G-cause ΔTB	1 Y	4.925	0.026	PM_2.5_ G-causes TB
	ΔTB does not G-cause ΔPM_2.5_	1 Y	0.578	0.447	TB does not G-cause PM_2.5_
VECM-based GC	PM_2.5_ does not G-cause TB	2 Y	2.490	0.114	PM_2.5_ does not G-cause TB
	TB does not G-cause PM_2.5_	2 Y	0.880	0.348	TB does not G-cause PM_2.5_
1997–2018 annual province-level data					
PVAR-based GC (cross-sectional)	PM_2.5_ does not G-cause TB	3 Y	W-Bar:7.57 Z-Bar:10.39	0.060	PM_2.5_ G-causes TB for at least one province
	TB does not G-cause PM_2.5_	3 Y	W-Bar:5.72 Z-Bar:6.18	0.214	TB does not G-cause PM_2.5_
2004–2018 monthly province-level data					
PVAR-based GC (cross-sectional)	PM_2.5_ does not G-cause TB	9 M	W-Bar:47.71 Z-Bar:50.80	< 0.001	PM_2.5_ G-causes TB for at least one province
	TB does not G-cause PM_2.5_	9 M	W-Bar:45.57 Z-Bar:48.00	< 0.001	TB G-causes PM_2.5_ for at least one province

VAR, vector autoregression model; Δ, 1st difference; G-cause, Granger-cause; GC, Granger causality test; VECM, vector error correction model; PVAR, panel vector autoregression model

Based on the panel data of from 1997 to 2018 (SI Appendix Table S3), the heterogeneous GC tests based on panel vector autoregressive model (PVAR) suggested unidirectional G-causality between PM_2.5_ and TB (Z-Bar 10.39, *P* = 0.060, Table 1, SI Appendix Table S4).

For the monthly data during 2004–2018 (SI Appendix Table S5), although pooled panel regression showed negative association between PM_2.5_ and TB incidence (SI Appendix Fig. S4), the meta-analysis of Pearson correlation coefficients (R) demonstrated positive association between them (overall R = 0.12, 95%CI 0.07–0.17, *P* < 0.001) (SI Appendix Fig. S5). The panel GC tests based on the cross-sectional Wald statistic suggested bidirectional G-causality between PM_2.5_ and TB (both *P* < 0.001) (Table 1, SI Appendix Table S6), although the converse scenario could not be true because TB cannot cause air pollution. This result was not surprising because the data duration was shorter and the threshold for rejecting the null hypothesis was causal relation in Granger sense for at least one province.

### CCM causal testing

**Fig. 2:**
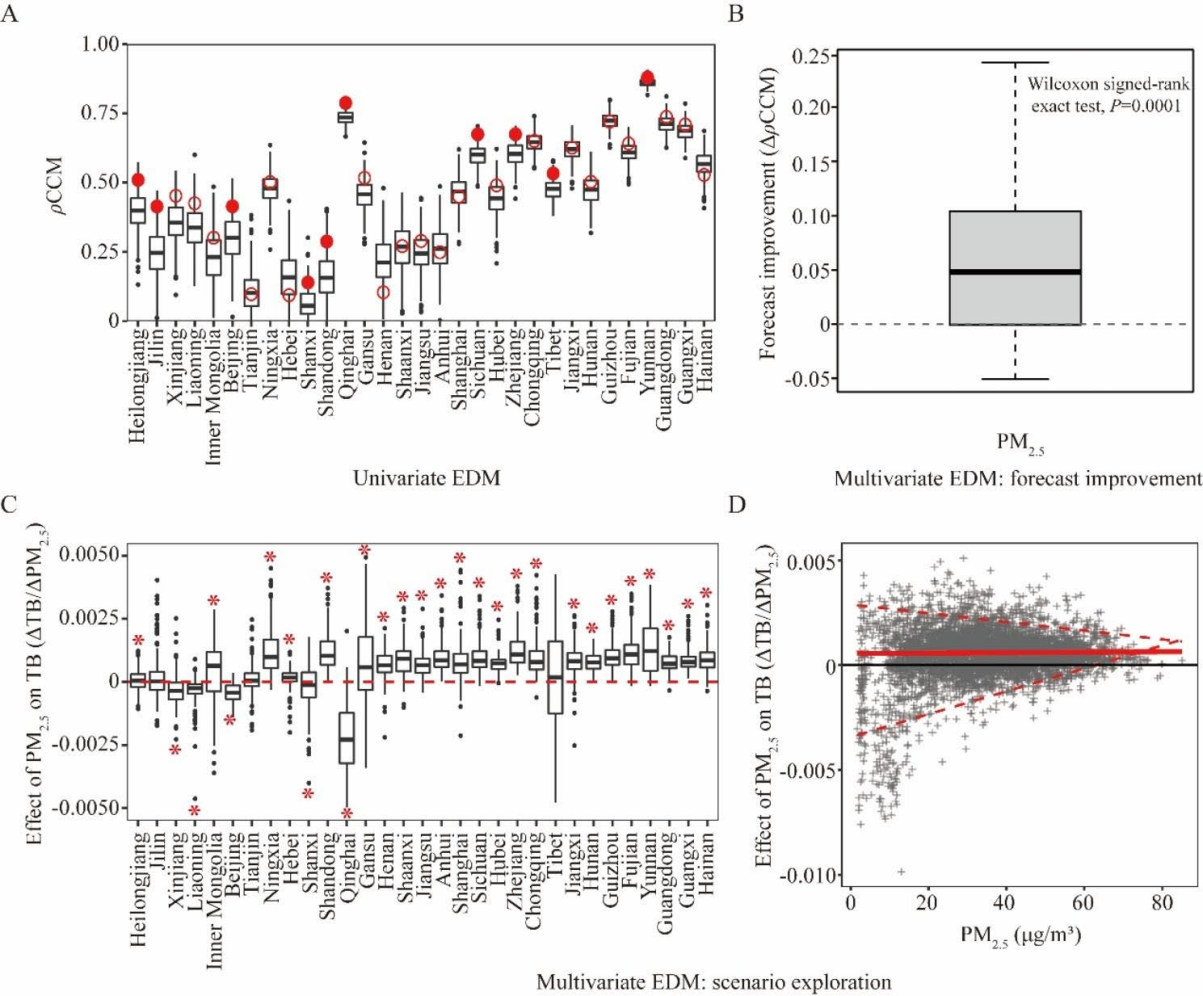
Cross-map causality of PM_2.5_ on tuberculosis. (**A**) Cross-map causality beyond shared seasonality of ambient PM_2.5_ on tuberculosis based on univariate SSR. The box-and-whisker plots show the null distributions for cross-map skill (ρCCM) expected from random surrogate time series which share the same seasonality as the true PM_2.5_ concentration. Red circles demonstrate the unlagged ρCCM for observed TB predicting purported PM_2.5_. Filled circles indicate the significant ρCCM (*P* ≤ 0.1). Provinces are ordered according to their latitudes. (**B**) Forecast improvement with multivariate SSR is quantified using ΔρCCM = ρCCM (with PM_2.5_) - ρCCM (without PM_2.5_). Wilcoxon signed-rank exact test reveals a significant difference. (**C**) Effect of PM2.5 on TB (ΔTB/ΔPM_2.5_) for each province. In the scenario analysis, PM_2.5_ shows a positive effect on TB incidence for 22 provinces (*P* ≤ 0.1). (**D**) Range of ΔTB/ΔPM_2.5_ as a function of PM_2.5_ grouped over all provinces. SSR, state-space reconstruction; CCM, convergent cross-mapping; ρCCM, the Pearson correlation between observations and CCM prediction

The seasonality of TB and PM_2.5_ was distinct at country level, with peaks in winter and spring respectively (SI Appendix Fig. S6). In addition, the heatmaps showed substantial temporal and geospatial variation of TB seasonality (SI Appendix Fig. S7). The mutual seasonality of TB and PM_2.5_ makes it especially important to distinguish causal interactions from spurious correlation. First, we performed univariate state-space reconstruction (SSR) with optimized CCM model parameters (SI Appendix Figs. S8, S9). The hypothesis was: if CCM prediction of TB for the observational PM_2.5_ was significantly better than it was for the null surrogates which had the same seasonal cycle as PM_2.5_ yet with randomized anomalies, the causal forcing of PM_2.5_ on TB would be established (SI Appendix Fig. S10). The box-and-whisker plot (Fig. 2A) demonstrated that PM_2.5_ be causal forcing for TB in 10 provinces, indicated by the measured cross-map skill (ρCCM) with significant *P* values (≤ 0.1). The results had very high metasignificance (Fisher’s method) for PM_2.5_: *P* < 4.2 × 10^− 5^. Second, we used the multivariate SSR to look for improvement in forecasting. That is, if the multivariate SSR containing the potential driving variable PM_2.5_ produced better forecasts of TB than without, then PM_2.5_ causally influenced TB in the CCM sense. It turned out that including PM_2.5_ led to significant improvement on forecast skill of TB (Fig. 2B). Third, we conducted scenario exploration with multivariate SSR. By predicting the change in TB (ΔTB) that result from a small change in PM_2.5_ (ΔPM_2.5_), we demonstrated that PM_2.5_ had a positive effect on TB incidence (positive values for ΔTB/ΔPM_2.5_) for 22 provinces individually (Fig. 2C) and for the whole group (Fig. 2D). Nevertheless, the combined results of the correlation and CCM analysis are provided in Table 2.

**Table 2:** Correlation and CCM causal analysis results between PM_2.5_ concentration and TB incidence across 31 provinces in China during 2004–2018

Province	Pearson correlation (R)	PM_2.5_ causes TB (ρ)	TB causes PM_2.5_ (ρ)	Causal direction^#^
Heilongjiang	0.0068	0.5101^*^	0.4036^***^	PM_2.5_↔TB
Jilin	0.1834^**^	0.4143^**^	0.0879	PM_2.5_→TB
Xinjiang	0.1722^**^	0.4527	0.1126	Neutrality
Liaoning	0.2709^***^	0.4256	0.3248	Neutrality
Inner Mongolia	0.1138	0.3014	0.0187	Neutrality
Beijing	0.2158^***^	0.4147^*^	-0.0413	PM_2.5_→TB
Tianjin	-0.0326	0.0977	-0.0166	Neutrality
Ningxia	0.2847^***^	0.5019	0.3743	Neutrality
Hebei	0.0321	0.0937	-0.2149	Neutrality
Shanxi	0.1552^**^	0.1397^**^	0.0913	PM_2.5_→TB
Shandong	0.0974	0.2879^*^	0.3084	PM_2.5_→TB
Qinghai	0.2644^***^	0.7879^***^	0.5165^*^	PM_2.5_↔TB
Gansu	0.3895^***^	0.5176	0.3645^**^	TB→PM_2.5_
Henan	-0.0090	0.1044	0.1777	Neutrality
Shaanxi	0.1264^*^	0.2712	0.1077	Neutrality
Jiangsu	0.0327	0.2896	0.4116^***^	TB→PM_2.5_
Anhui	0.0789	0.2491	0.3672^**^	TB→PM_2.5_
Shanghai	0.0102	0.4501	0.1720	Neutrality
Sichuan	0.1928^***^	0.6745^**^	0.4451^*^	PM_2.5_↔TB
Hubei	0.0619	0.4903	0.4764^**^	TB→PM_2.5_
Zhejiang	0.0716	0.6748^**^	0.4381^***^	PM_2.5_↔TB
Chongqing	0.1054	0.6511	0.4271	Neutrality
Tibet	0.4404^***^	0.5331^*^	0.3843	PM_2.5_→TB
Jiangxi	0.0736	0.6263	0.3711	Neutrality
Hunan	0.0165	0.5033	0.6260^***^	TB→PM_2.5_
Guizhou	0.1276^*^	0.7201	0.5867^*^	TB→PM_2.5_
Fujian	0.1479^**^	0.6415	0.1869	Neutrality
Yunnan	0.1828^**^	0.8796^*^	0.4377	PM_2.5_→TB
Guangdong	-0.0493	0.7374	0.6069^***^	TB→PM_2.5_
Guangxi	0.0227	0.7098	0.5783^**^	TB→PM_2.5_
Hainan	-0.0929	0.5281	0.4971^**^	TB→PM_2.5_

^*^P ≤ 0.1, ^**^P ≤ 0.05, ^***^P ≤ 0.01

^#^The converse scenario could not be true because tuberculosis does not cause air pollution, that is, the shadow attractor constructed using PM_2.5_ data should not contain information to accurately reconstruct past TB incidence

### The exposure–response effects of air pollutants on TB risk

**Fig. 3:**
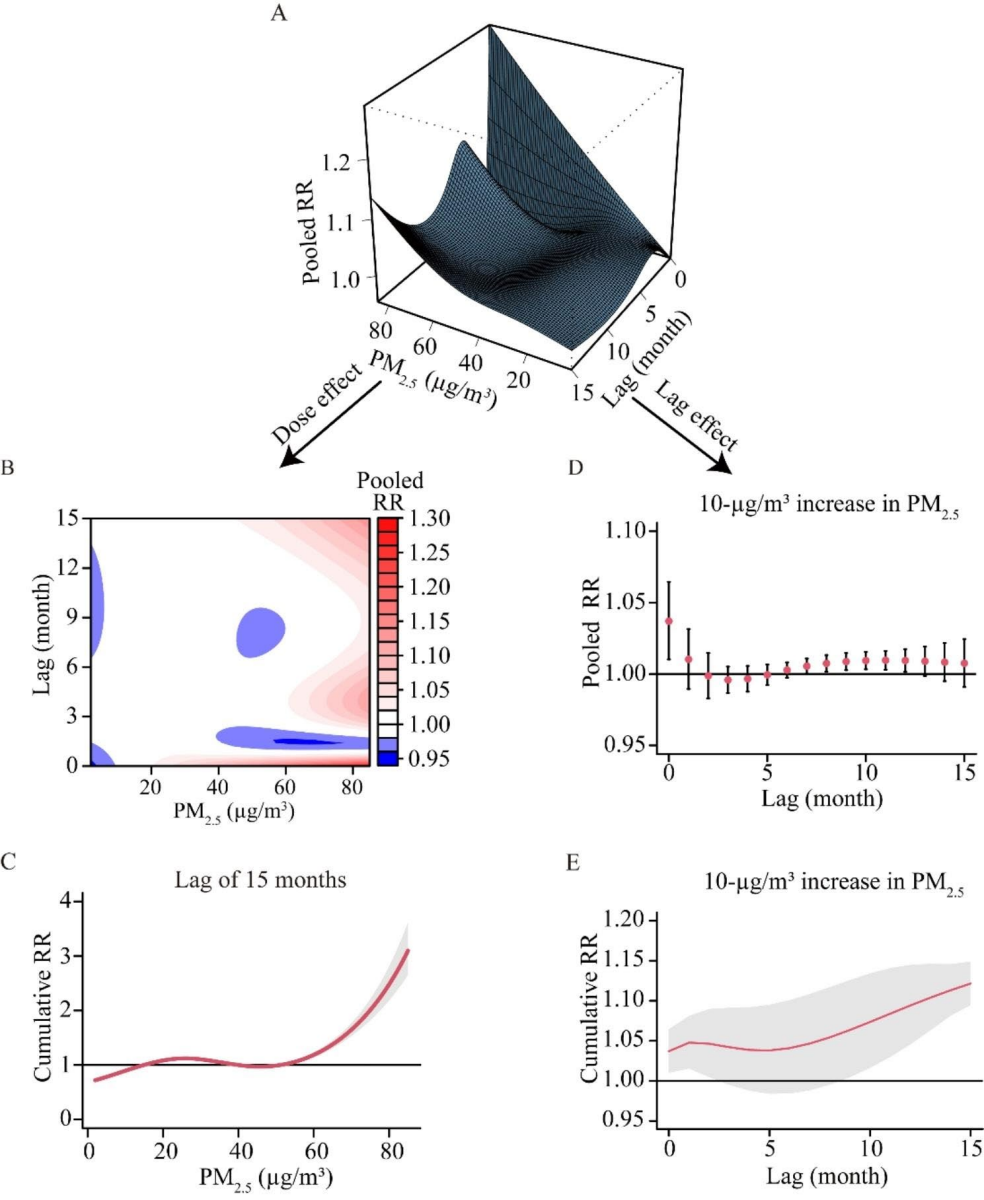
Exposure-response relationship between PM_2.5_ and tuberculosis incidence in single-pollutant DLNM model. (**A**) Three-dimensional plot: the height of the hexahedron represents RR for the association between TB incidence and ambient PM_2.5_ exposure, while two bottom edges represent the full range of monthly mean PM_2.5_ concentration and the number of months delayed. (**B**) Contour plot: the red color gradient represents RR > 1, and the blue gradient represents RR < 1. (**C**) Cumulative effects of PM_2.5_ exposure for 15 months. (**D**-**E**) Pooled and cumulative effects with 10 µg/m^3^ increase in PM_2.5_ throughout 0–15 months. The reference level of PM_2.5_ is set as 15 µg/m^3^. Monthly mean temperature, precipitation and sunshine duration, and annual population density, GDP per capita, certified doctors and beds of medical institutions are added as time-varying local control variables. TB, tuberculosis; RR, relative risk

Based on the multivariate DLNM model, the three-dimensional graph vividly depicted the overall effects of PM_2.5_ on TB incidence, calculated as relative risks (RRs) (Fig. 3A). In the contour plot, acute effects (lag 0–1 months) were observed under exposure to high levels of PM_2.5_ (with the maximum pooled [lag-specific] RR of 1.28 under exposure to 85 µg/m^3^ of PM_2.5_ at the current month), while delayed effects were seen under exposure to high levels of PM_2.5_ at lag 2–15 months (Fig. 3B). The cumulative (15 months) effects of PM_2.5_ on TB incidence were demonstrated in the exposure-response curve (Fig. 3C). Besides, the pooled and cumulative (throughout lags of 0–15 months) RRs associated with 10-µg/m^3^ increase in PM_2.5_ were shown in Fig. 3D and E respectively.

The pooled exposure–response effects of air pollutants on TB risk in both the single-pollutant and two-pollutant models were shown in Table 3. In single pollutant model, each 10 µg/m^3^ increase in PM_2.5_ concentrations was significantly positively associated with the TB incidence, with RR of 1.121 (95% CI:1.095, 1.149). Moreover, there was no substantial change in the results when conducting the multi-pollutant models.

**Table 3:** Cumulative association between tuberculosis incidence and 10 µg/m^3^ increase in PM_2.5_.

Model	Pollutant(s)	RR (95%CI) for TB (associated with 10 µg/m3 increase in PM_2.5_)
Single-pollutant	PM_2.5_	1.121 (1.095, 1.149)^*^
Multi-pollutant	PM_2.5_ + PM_10_	1.209 (1.149, 1.273)^*^
	PM_2.5_ + CO	1.112 (1.085, 1.139)^*^
	PM_2.5_ + O_3_	1.292 (1.257, 1.327)^*^
	PM_2.5_ + PM_10_ + CO	1.267 (1.189, 1.350)^*^
	PM_2.5_ + PM_10_ + O_3_	1.208 (1.144, 1.277)^*^
	PM_2.5_ + CO + O_3_	1.307 (1.271, 1.344)^*^
	PM_2.5_ + PM_10_ + CO + O_3_	1.183 (1.108, 1.263)^*^

TB, tuberculosis; PM_2.5_, particulate matter of < 2.5 μm; PM_10_, particulate matter of < 10 μm; CO, carbon monoxide; O_3_, ozone. ^*^P ≤ 0.05

## Discussion

Evaluating the influence of PM_2.5_exposure on TB occurrence holds substantial relevance in the realm of public health, serving as the initial phase in formulating environmental strategies aimed at alleviating the tuberculosis burden within the context of China. Several empirical studies have addressed the potential relation between air pollution and TB incidence, but this issue remains controversial and inconclusive, deserving further investigation [31]. The information presented here refers to situations within China, but environmental health and protection are known without boundaries. The causal inference framework may be valuable for the identification of other air pollution-associated adverse health impacts.

Ambient air pollution is one of the leading environmental risk factors to human health. Short-term air pollution exposure is found to be causally related to acute adverse respiratory health effects and exacerbation of preexisting chronic airway diseases, while long-term exposure may be a causal factor for new-onset airway diseases such as childhood asthma [32]. PM_2.5_ (also called alveolar fraction) accounts for 96% of particles observed in human pulmonary system [33]. The toxicity of PM is inversely linked to particle size, with smaller particles contributing to greater inflammatory effects [34]. There are biological mechanisms by which PM_2.5_ could plausibly affect individual’s susceptibility to TB infection or reactivation. First, PM_2.5_ could directly attack the respiratory tract and suppress antimicrobial activity by down-regulating airway antimicrobial proteins and peptides (AMPs) which are important for airway innate immunity [35]. Second, it may disrupt the synthesis and secretion of inflammatory cytokines and impair anti-mycobacterial T cell immune responses to *M tb* [36]. Third, increased iron availability provided by PM_2.5_ may create a favorable environment for mycobacterial proliferation [37, 38]. Based on the above, PM_2.5_ served as the best indicator of all air pollutants here. Our findings support that exposure to air pollutants above a certain level may increase their susceptibility to *M. tb* infection or reactivation.

Two earlier cohort studies reported potential association between PM_2.5_ and TB in Los Angeles city, USA and Taiwan province, China respectively [39, 40]. The results from time series studies on this issue contradicted one another. The inconsistent evidence may partly be due to the different methods, variable selection and time frames. A recent meta-analysis claimed that PM_2.5_ had neither long-term nor short-term TB risk (RR, 1.030; 95%CI, 0.996–1.065 and RR, 1.031; 95%CI, 0.981–1.083 respectively) [31]. However, this study argues that, the existing studies were restricted to a partial view of the phenomenon. In this respect, our study departs from the literature by taking into consideration the information from both the province (piece) and country (whole puzzle) sides, relative to their characteristics, heterogeneous settings and common trend. To do so, we analyzed the data from 31 provinces in China. Our findings could be convincing given the country’s sheer size and the allowance for temporal diversity.

Moving beyond correlation, we evaluated the causality between PM_2.5_ and TB with complementary strategies. To determine whether X causes Y: GC compares “knowledge about Y_t_” vs. “knowledge about X_t_ and Y_t_” in prediction of Y_t+1_ (forward looking) [41], while CCM compares “knowledge about M_Y_” vs. “no knowledge about M_Y_” in prediction of X_t_ (backward looking) [27]. GC can perform relatively well on short time series, while CCM generally prefer for longer time series (≥ 30 observations) [25]. The two seemingly opposite methods can yield similar causal inference in spite of the different assumptions [13]. Herein, GC or CCM (or both) were decided according to the aims and data characteristics, rather than “linear vs. nonlinear model” gradient. We re-enforced the causal effect of PM_2.5_ on TB by employing GC and CCM on the long panel dataset. Our approach has an advantage over the standard approach based on regression as it is free from issues concerning the exposure-confounders-morbidity modeling and does not involve extrapolation.

It is worth noting that, from exposure-response relationship perspective, PM_2.5_ was positively associated with both TB incidence, with RR of 1.12 (95% CI: 1.03, 1.22) per 10 µg/m^3^ increase, which was consistent with our results [42]. A regional study also demonstrated that long-term exposure to PM_2.5_ was significantly associated with higher TB incidence [43]. Increased exposure to PM_2.5_ contributed to a faster bacterial replication rate, indicating that *M. tb* exhibits increased reproductive activity, thus accelerating within-host endogenous reactivation [44]. Elevated concentrations of PM_2.5_ may exert pressure on healthcare systems through an augmentation in TB incidence and associated treatment expenditures.

During 2006–2012, China’s new air pollution policies which interact with political incentives were introduced in the 11th Five-Year Plan. These policies have been effective in cutting pollutants emission. After the winter-long “PM_2.5_ crisis” in eastern China in 2013, the standards for air pollution control have been updated and further strengthened [45]. The observed co-movement between PM_2.5_ and TB incidence suggest a possible link between the air pollution control policies and health risk reduction. Therefore, TB prevention should not only focus on interrupting TB transmission, but also on monitoring air pollutants such as PM_2.5_. Establish real-time air quality monitoring systems to notify the public and policymakers of elevated pollution levels, encouraging precautionary measures. Allocate healthcare resources efficiently in regions with significant burdens of TB and elevated PM_2.5_ levels.

This study has several limitations. First, the estimate for exposure-response relationship should be interpreted with caution. It cannot be extended to concentrations beyond the support of the data. Second, the effect of air pollution control policies on TB has not been tested. The counterfactual models such as difference-in-differences (DID) may be helpful for policy evaluation. Third, although the effects of PM_2.5_ to drive TB may be different for new infection and reactivation, we could not test the hypothesis. It is usually difficult to judge whether an active TB case is from LTBI or uninfected individuals in routine practice. Last, we analyzed the impacts of PM_2.5_ at the province level, yet different cities and counties might be heterogeneous even within one province. Prospective spatially oriented causal research endeavors have the potential to yield novel insights for elucidating heterogeneity.

In summary, we demonstrate that ambient PM_2.5_ exposure and tuberculosis incidence had a linkage which (1) is causal and ecologically important; (2) is independently detected in different provinces; and (3) follows an exposure-response gradient. The take-home message is clear: to fight tuberculosis, we must also fight air pollution.

### Electronic supplementary material

Below is the link to the electronic supplementary material.

Supplementary Material 1

## Data Availability

The datasets supporting the conclusions of this article are available in the Data-center of China Public Health Science, http://www.phsciencedata.cn/Share/en/index.jsp. The codes are available at 10.5281/zenodo.10020179.
