# Supplementary material for: Population impact of fine particulate matter on tuberculosis risk in China: a causal inference
Source: BMC Public Health. 2023 Nov 18;23:2285. doi: 10.1186/s12889-023-16934-8 (PMC10657490; doi:10.1186/s12889-023-16934-8)
Supplement: Supplementary file 1 — Supplementary Material 1 [file 12889_2023_16934_MOESM1_ESM.docx]

**Supplementary Information for:**

**Population impact of fine particulate matter on tuberculosis risk in China: A causal inference**

**This PDF file includes:**

Extended Methods

Fig. S1-S10

Table S1-S6

Extended References

**Extended Methods**

**Data source**

The time series data of annual reported number of pulmonary tuberculosis (PTB) in China during 1982-2019 was collected from online global TB database (<https://worldhealthorg.shinyapps.io/tb_profiles/>) released by the World Health Organization (WHO) [1]. The panel data of TB incidence in 31 provinces (annually during 1997-2018, and monthly during 2004-2018), was obtained from Chinese public health science data center (<https://www.phsciencedata.cn/>) based on China’s web-based national infectious disease reporting system (NIDRS). NIDRS collected case-level data on 40 notifiable infectious diseases from almost all healthcare facilities across the country [2]. HIV-associated TB cases were not included for this analysis.

The air pollutant and weather data were retrieved from the modern-era retrospective analysis for research and applications version 2 (MERRA-2) released by national aeronautics and space administration (NASA) of USA [3]. The satellite-based data has been validated with ground-based data in China [4]. The MERRA-2 data are available on its 0.5° latitude × 0.625° longitude grid level for each month since 1980. We resampled the data to the 0.1° × 0.1° grid level using “interp.surface” bilinear interpolation function in “fields” package of R software. The grids were aggregated to county level (2900 counties) for each month and further averaged to provincial level (31 provinces). The concentration of fine particulate matter (PM_2.5_) was calculated using surface mass concentrations of five PM species according to the adopted Equation 1 [4, 5].

[PM_2.5_] (kg/m^3^) = [BC] +1.6×[OC] + 1.375×[SO_4_] + [DUST_2.5_] + [SS_2.5_]

(Equation 1)

where,

BC: black carbon;

OC: organic carbon;

SO4: sulfate ion;

DUST_2.5_: dust with diameters less than 2.5 μm;

SS_2.5_: sea salt with diameters less than 2.5 μm.

The meteorologic data on monthly average air temperature (℃), specific humidity (g/kg), pressure (Pa), cumulative sunshine duration (hour) and precipitation (mm) was retrieved from MERRA-2 using the same method. Relative humidity was calculated from specific humidity, temperature and pressure with the “SH2RH” function in the “humidity” package of R.

The national and provincial-level data on annual birth rate, population size, and per capita GDP were extracted from the governmental statistical yearbooks (<http://www.stats.gov.cn/tjsj/ndsj/>).

Institutional board review was waved because no individual-level data was used.

**Granger causality (GC) tests**

**Time series GC tests**. The granger analysis was performed to explore the causal relationship between PM_2.5_ and TB with the 1982-2019 time series data. As preliminary analysis, Kwiatkowski, Phillips, Schmidt and Shin (KPSS) and augmented Dickey–Fuller test based on GLS-detrending (DF-GLS) tests were utilized to verify the stationarity of the series through hypotheses. If the time series had a unit root, a difference in the series was applied to make it stationary and the vector autoregressive (VAR) models were applied in terms of their first differences (Equation 2) [6]. When both series were determined I(1) and co-integrated, vector error correction (VECM) models provided an alternative option [7]. After selecting the optimal lag order using the Akaike information criterion (AIC), Bayesian information criterion (BIC) and Hannan-Quinn information criterion (HQIC) methods [8], the VAR and VECM models were analyzed with the maximum likelihood method estimation (MLE). Through the use of the impulse response function and the variance decomposition, the models showed the contemporary relationship among the variables and help to explain their dynamics. Finally, The Granger causality Wald F test was applied to calculate the significance of G-causality.

$${dTB}_{t}=\alpha_{1}+\sum_{k=1}^{K} {\beta_{1k}dTB}_{t-k}+\sum_{k=1}^{K} {\gamma_{1k}dPM}_{t-k}+\varepsilon_{1t}$$

$${dPM}_{t}=\alpha_{2}+\sum_{k=1}^{K} {\beta_{2k}dTB}_{t-k}+\sum_{k=1}^{K} {\gamma_{2k}dPM}_{t-k}+\varepsilon_{2t}$$

(Equation 2)

where,

d*TB*: TB incidence (first difference);

d*PM*: PM_2.5_ concentration (first difference)

*α*：contant;

*β, γ*: autoregressive coefficient;

*k*: lag length (k=1,…, K)；

*ε_it_*: random error term (white noise disturbances).

Regarding causality within this system, there is one-way causality running from PM to TB if not all γ_1k_'s are zero but all β_2k_'s are zero.

**Panel GC tests.** The GC analysis was also applied to 1997-2018 annual panel data and 2004-2018 monthly panel data. First, Levin-Lin-Chu (LLC) method was used to test the presence of the panel unit root. If no variable contained panel unit root, the panel VAR models (Equation 3) would be applied. Second, Frees tests were performed to detect the presence of cross-sectional dependence. As the variables may have spatial spillover effects, ignoring cross-sectional dependence would lead to inaccurate estimates. The solution relies on Monte-Carlo or Bootstrap simulation [9]. Third, the optimal lag orders were chosen using the information criteria. Last, panel GC tests based on group mean Wald-statistic were performed.

$${TB}_{it}=\theta_{i}+\sum_{k=1}^{K} {\beta_{i}^{(k)}TB}_{i,t-k}+\sum_{k=1}^{K} {\delta_{i}^{(k)}PM}_{i,t-k}+\varepsilon_{it}$$

(Equation 3)

where,

*TB*: TB incidence (stationary variable) observed for provinces i in year t;

*PM*: PM_2.5_ concentration (stationary variable);

*θ*：fixed individual effects;

*β*: autoregressive coefficient;

δ: regression coefficients;

*k*: lag length.

The heterogenous panel GC test is based on the null hypothesis of homogeneous non-causality: there is no causal relationship from *PM* to *TB* for all the provinces of the panel. Under the alternative hypothesis, there exists a causal relationship from *PM* to *TB* for at least one province of the sample.

The analyses were performed using the standard modules (e.g., var, vec, vargranger, xtgcause) in Stata 17.0 (StataCorp, Texas, USA).

**Meta-analysis of correlation coefficients**

Pearson correlation coefficient (R) between PM_2.5_ concentration and TB incidence from 31 provinces were used to create the summary estimates. The R values were converted to a standard normal metric (Fisher's Z) for overall effect size calculation through random effects models. The pooled Fisher’s Z values and their 95% confidence intervals (CIs) were then transformed back into correlation R values for presentation [10]. The analyses were conducted with Stata 17.0 (StataCorp, Texas, USA).

**Convergent cross mapping (CCM) method**

We used empirical dynamic modeling (EDM), a data-driven, equation-free, mechanistic approach [11], to model mechanisms forcing TB epidemics. Convergent cross-mapping (CCM) method was adopted to distinguish causality between pairs of time series from correlations. The basic idea of CCM is to look for the signature of X in Y’s time series. In brief, two manifolds are constructed from lagged coordinates of X and Y (Equation 8). An algorithm was constructed with two time series of PM_2.5_ (X) and TB (Y): X｛x_1_, x_2_,…x_L_｝Y｛y_1_, y_2_,…y_L_｝(L, length). The shadow manifolds of M_X_ and M_Y_ were reconstructed (so called state-space reconstruction, SSR). If the attractor recovered from manifold M_Y_ is able to predict the states of variable X, we could say that variable X “CCM-causes” Y [12].

x(t) = <X_t_, X_t-τ_, X_t-2τ_, …X_t-(E-1)τ_>

y(t) = <Y_t_, Y_t-τ_, Y_t-2τ_,…Y_t-(E-1)τ_>

(Equation 8)

where,

τ: time lag;

E: embedding dimension.

We assessed the CCM causality between PM_2.5_ and TB incidence based on univariate state-space reconstruction (SSR) according the modified methods described elsewhere [13, 14]. First, using simplex projection, we determined the embedding dimension (E). Simplex projection is a nearest-neighbor-based nonlinear regression performed on an attractor to determine whether the system is forecastable beyond the skill of an autoregressive model [15]. We chose the best E that gave highest predictability, ensuring that E was sufficiently large to capture the dynamics of the system without including extraneous dimensions. The time lag τ was set to 1, which is the smallest temporal resolution in the created delay time series. Second, we performed S-map tests on the two time series for nonlinearity and systematic noise. Third, we calculated the CCM correlation between PM_2.5_ and TB, i.e., the two variables’ abilities to describe each others’ dynamics. Fourth, for each variable, we created 500 surrogates time series that had the same seasonal average, but with random anomalies. Then we tested whether cross-mapping prediction was significantly better for the real observational time series than it was for the surrogate time series. Sixth, we examined whether the cross-map prediction skill increased and demonstrated convergence as the library length increased if two variables existed causality. CCM for the real time series need to show higher prediction skill (ρ) than 90% confidence intervals of surrogate time series. Last, we determined the metasignificance of the CCM tests using Fisher’s method, which relied on the assumption of the independent *P* values.

A univariate embedding with dimension E < E^*^ does not contain full information about the system state and dynamics (so called “under embedded”) [13]. Therefore, as an additional test for causality, multivariate SSR (including stochastic causal variables as a coordinate in the state space) could improve the ability of nearest-neighbor prediction. For seasonal TB, PM_2.5_ could be considered stochastic because information about it may already be included in the univariate embedding.[11] Hence, according to a modified method developed by a previous study [14], we examined multivariate SSR forecast improvement. Significance of improvement is determined using the Wilcox test.

Scenario exploration with multivariate SSR was employed to investigate the effect of a small change in the potential driver (PM_2.5_) on TB incidence across different states of the system. Before scenario exploration, we normalized the data on monthly TB incidence through dividing the annual incidence in the province (averaged over all years included). We treated 5% of the variance for PM_2.5_ in all provinces as the value of ΔPM_2.5_. The S-map tests were applied at each time step t to forecast TB incidence three months later, with a small increase (+ΔPM_2.5_/2) and a small decrease (−ΔPM_2.5_/2) of the observed value of PM_2.5_. By calculating the change in TB dynamics which results from a small change in driving variable, we estimated the local effect PM_2.5_ has on the TB incidence. It is worth noting that ρCCM derived from univariate SSR gave the significance of causality without telling whether the influence was positive or negative. Therefore, the effect of ΔTB/ΔPM_2.5_ provided a way to understand the causality direction.

The analyses were performed using rEDM package version 0.7.5 of R software (R Foundation for Statistical Computing, Vienna, Austria).

**Distributed lag nonlinear model (DLNM)**

The basic model of DLNM is generalized linear model (GLM). Based on DLNM we estimated the exposure-response relationship between PM_2.5_ and TB incidence. The cumulative relative risks (RRs) were calculated for different extents of exposure to PM_2.5_ within lag 0-15 months, as well as for every 10 μg/m^3^ of PM_2.5_. The reference values of PM_2.5_ was set as 15 μg/m^3^ according to WHO’s air quality guidelines (<https://www.who.int/publications/i/item/9789240034228>). In the multivariate DLNM, temperature, precipitation and sunshine duration were included to control the potential confounders. We adjusted the temporal trend and incidence in the previous month in the models. Moreover, a natural cubic spline (“ns”) function was adopted to control the weather factors, seasonal variation, and long-term trends [16].

In order to fit the nonlinear and delayed effects, we constructed “cross-basis” (bidimensional) function and depicted the effects of predictors and lags simultaneously. Moreover, we computed a three-dimensional model of PM_2.5_, lag months and risk of TB incidence into a hexahedron.

The analyses were performed using the package “dlnm” version 2.4.7 in R software (R Foundation for Statistical Computing, Vienna, Austria).

**Data Availability**

The data that supports the findings of this study are available in the supplementary material.

**Figure S1**


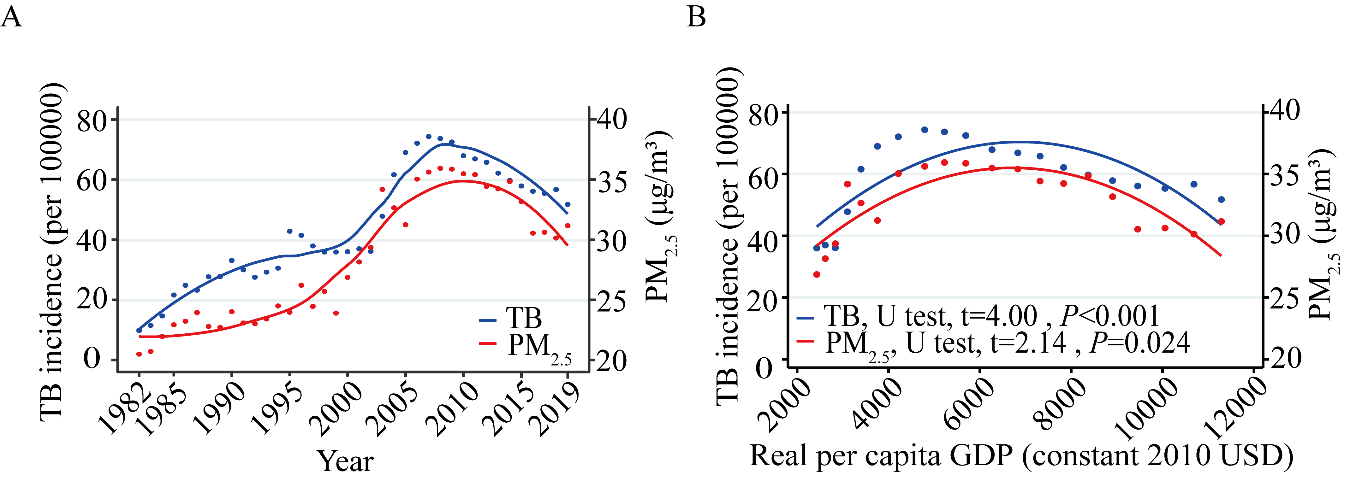


**Figure S1**. Correlation of TB incidence, PM_2.5_ concentration and real per capita GDP in China.

**Figure S2**


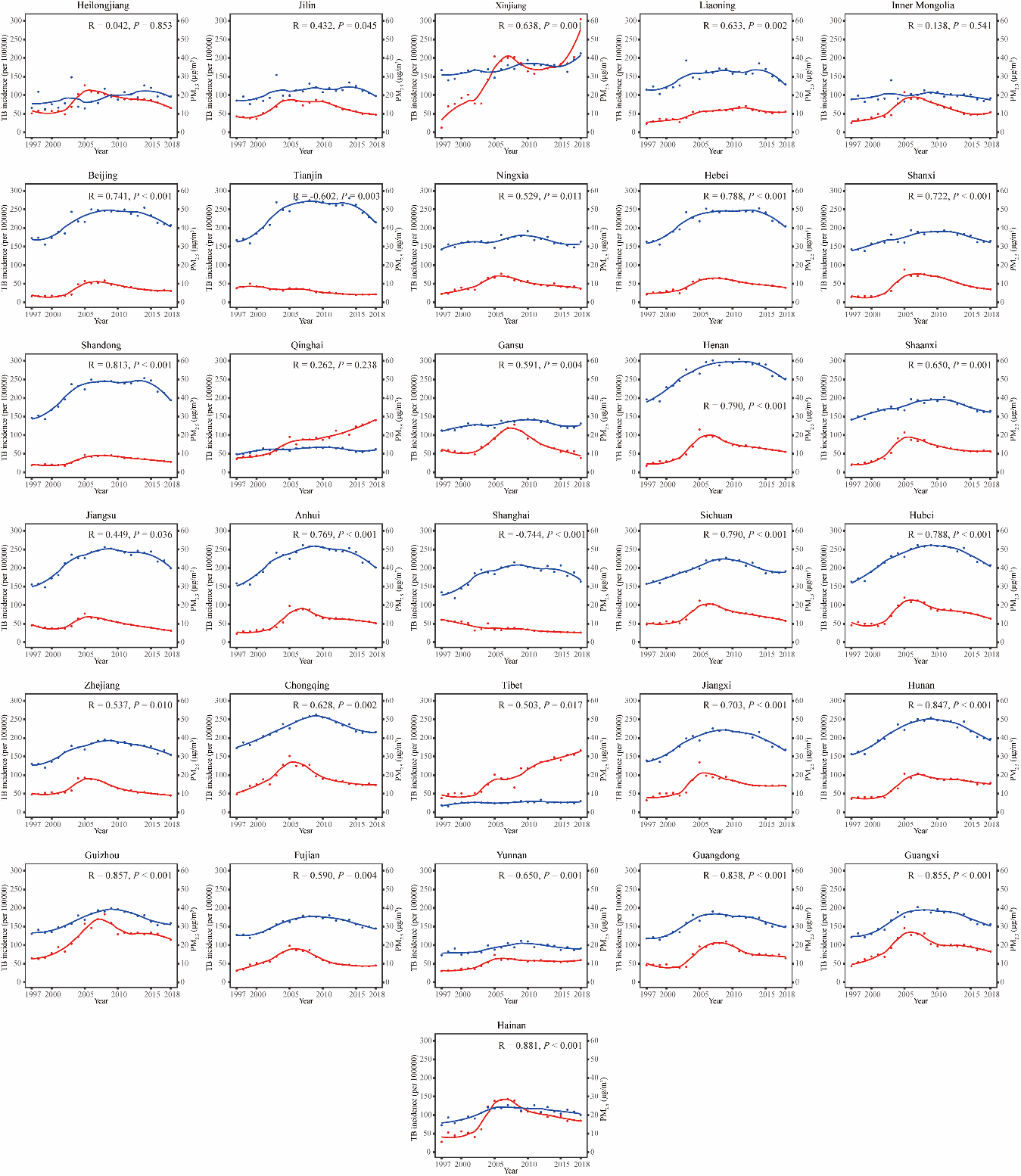


**FigureS2.** Correlation analysis between PM_2.5_ concentration and TB incidence in 31 provinces, 1997-2018.

**Figure S3**

**
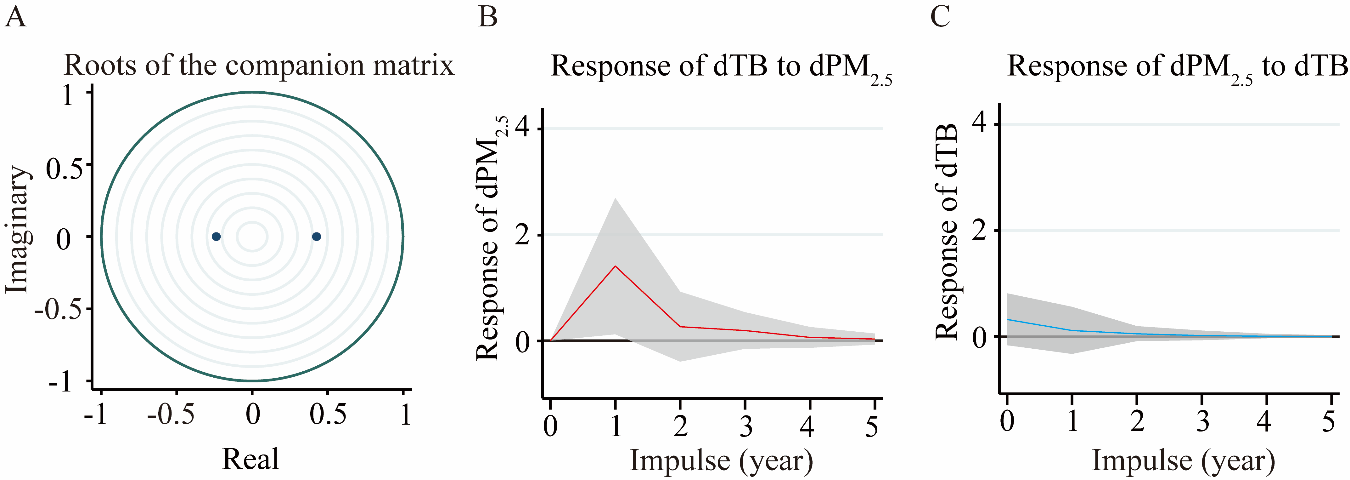
**

**Figure S3.** Stability and impulse-response function of vector autoregressive (VAR) model.

(A) VAR model stability test. All roots of AR characteristics polynomial of the series lie inside the unit circle. (B) Response of differenced TB to impulse of differenced PM_2.5_. (C) Response of differenced PM_2.5_ to impulse of differenced TB.

**Figure S4**

**
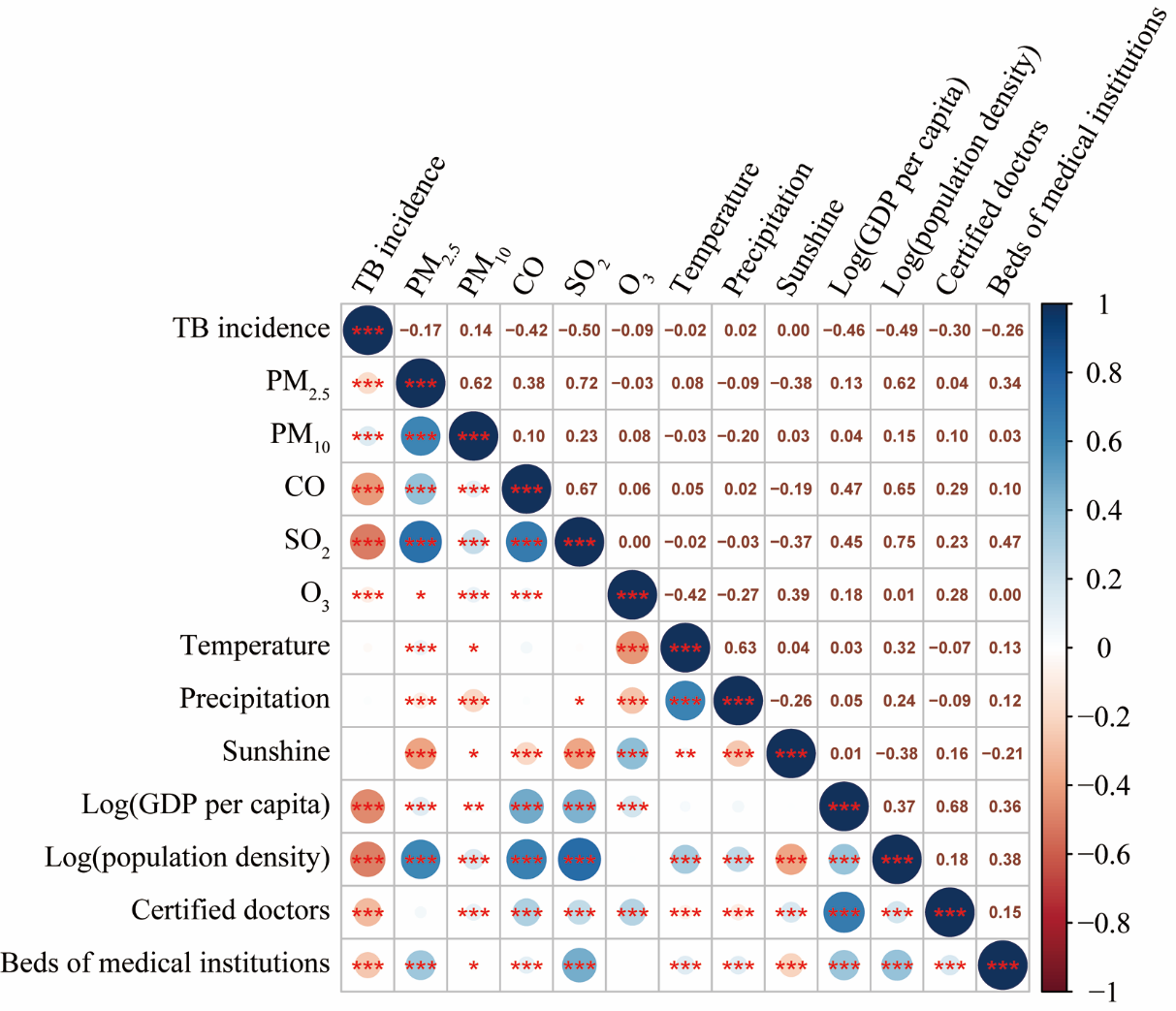
**

**Figure S10**. Pearson correlation matrix of monthly TB incidence, PM_2.5_ concentration, weather and socio-economic covariates in 31 provinces of China during 2004-2018.

The values are the pairwise correlation coefficients (r), with blue and red color indicating statistically significant positive and negative correlation (*P* ≤ 0.05) respectively.

^*^ *P* ≤0.05； ^**^ *P* ≤ 0.01；^***^ *P* ≤ 0.001.

**Figure S5**

**
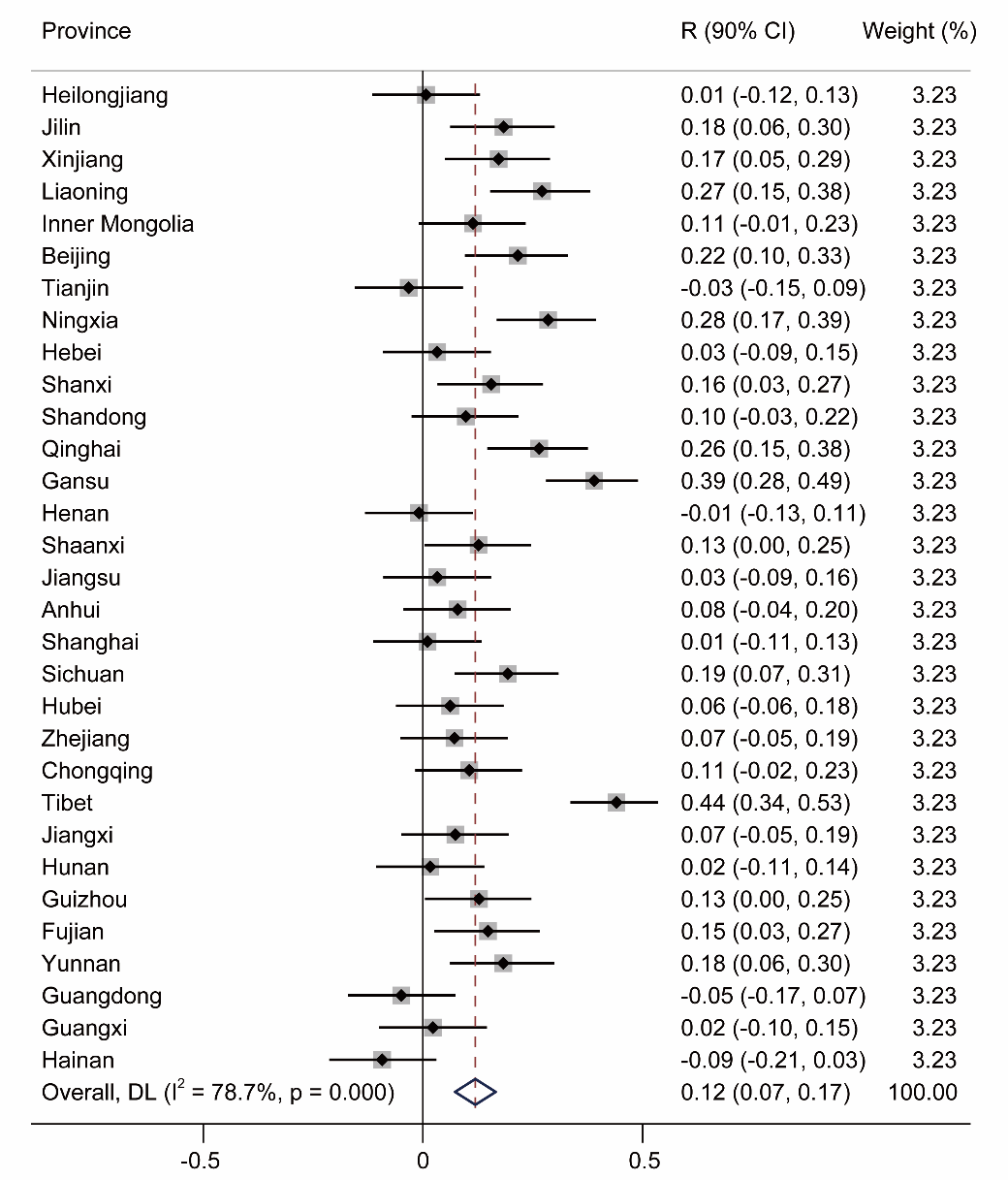
**

**Figure S5**. Association between the PM_2.5_ concentration and TB incidence in 31 provinces in mainland China, 2004-2018.

Forest plot detailing the summary Pearson correlation coefficients (R) and corresponding 95% CIs.

**Figure S6**


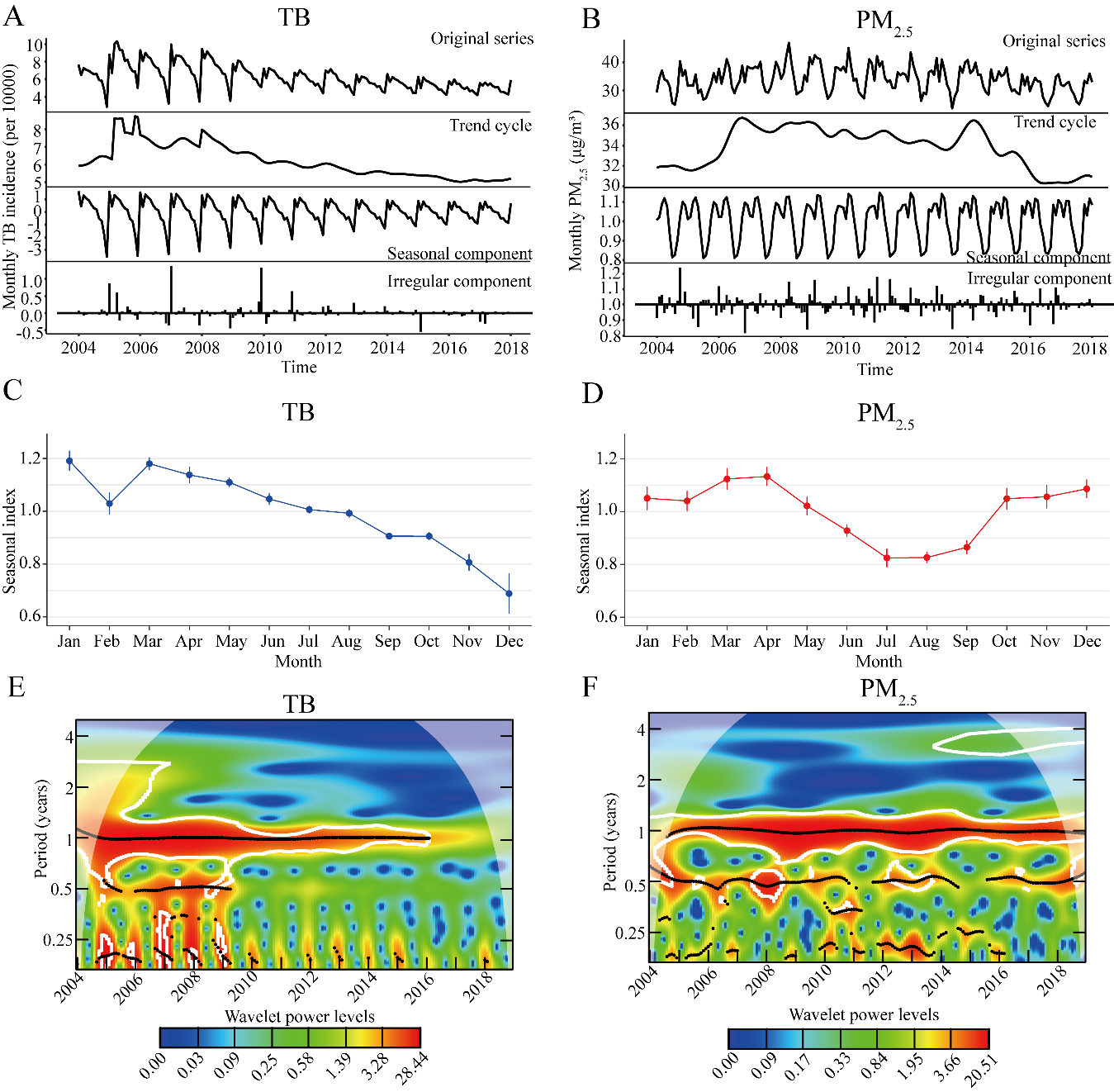


**Figure S6**. Seasonality of TB incidence and PM_2.5_ in China during 2004-2018.

(A, B) Using X-12-ARIMA program, the original time series of TB and PM_2.5_ are decomposed into three basic components: trend cycle, seasonal and irregular. (C) The seasonal index for TB ranged from 0.67 to 1.19. The annual epidemic peak was observed from Jan to Jun. (D) The seasonal index for PM_2.5_ ranged from 0.82 to 1.13. The annual epidemic peak was observed from Oct to May. (E, F) Wavelet power spectrum for the time series of TB incidence and PM_2.5_, where red areas represent strong evidence for seasonality at that time point (x-axis) and inter-peak period (y-axis).

**Figure S7**

**
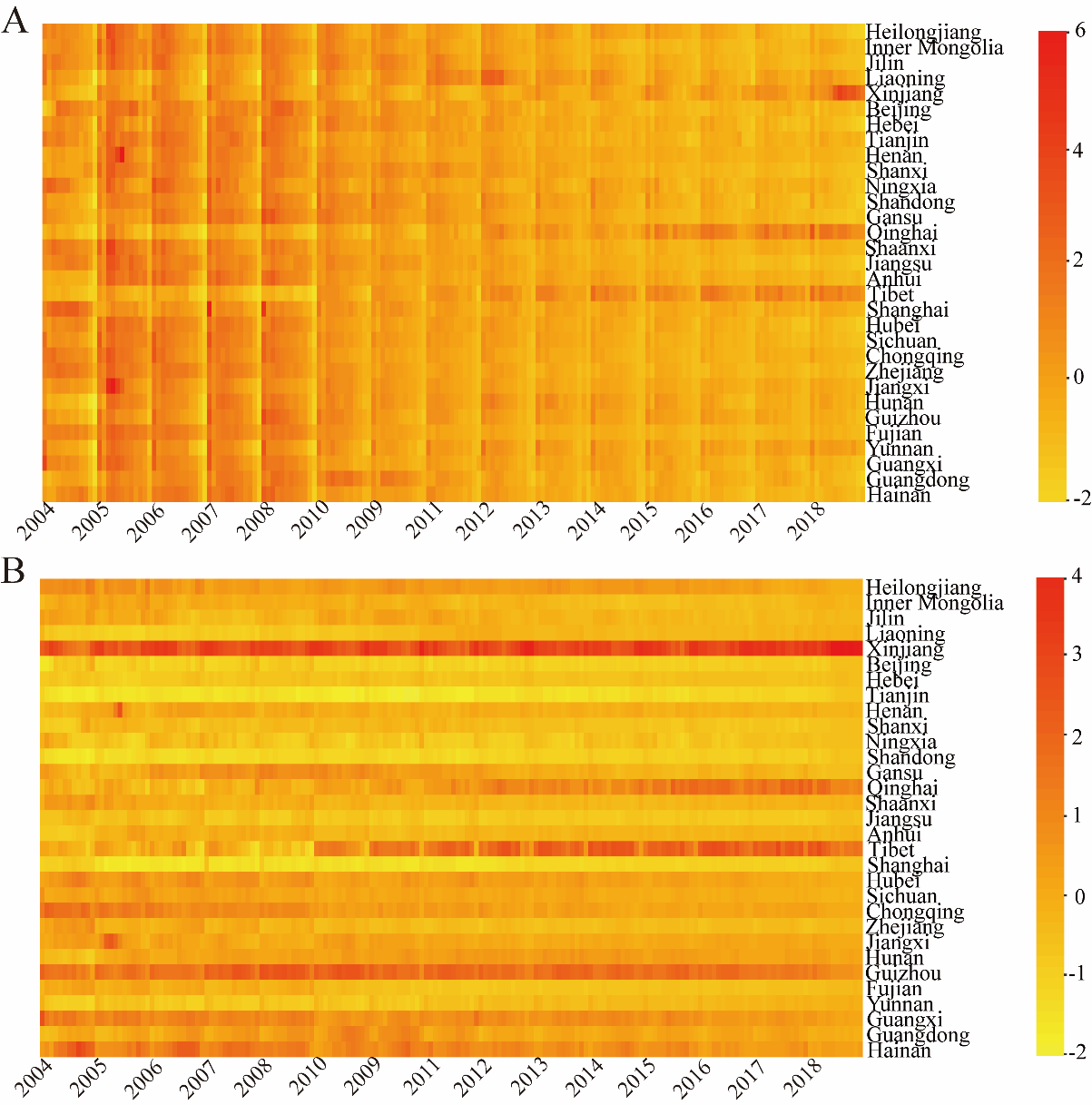
**

**Figure S7**. Heatmaps of TB incidence for 31 provinces in China during 2004-2018.

(A) TB incidence normalized per province to show temporal variation. (B) TB incidence normalized per month to show geographical variation.

Each horizontal bar represents a province, ordered by latitude. Each vertical bar represents one month over the 15-year period.

**Figure S8**


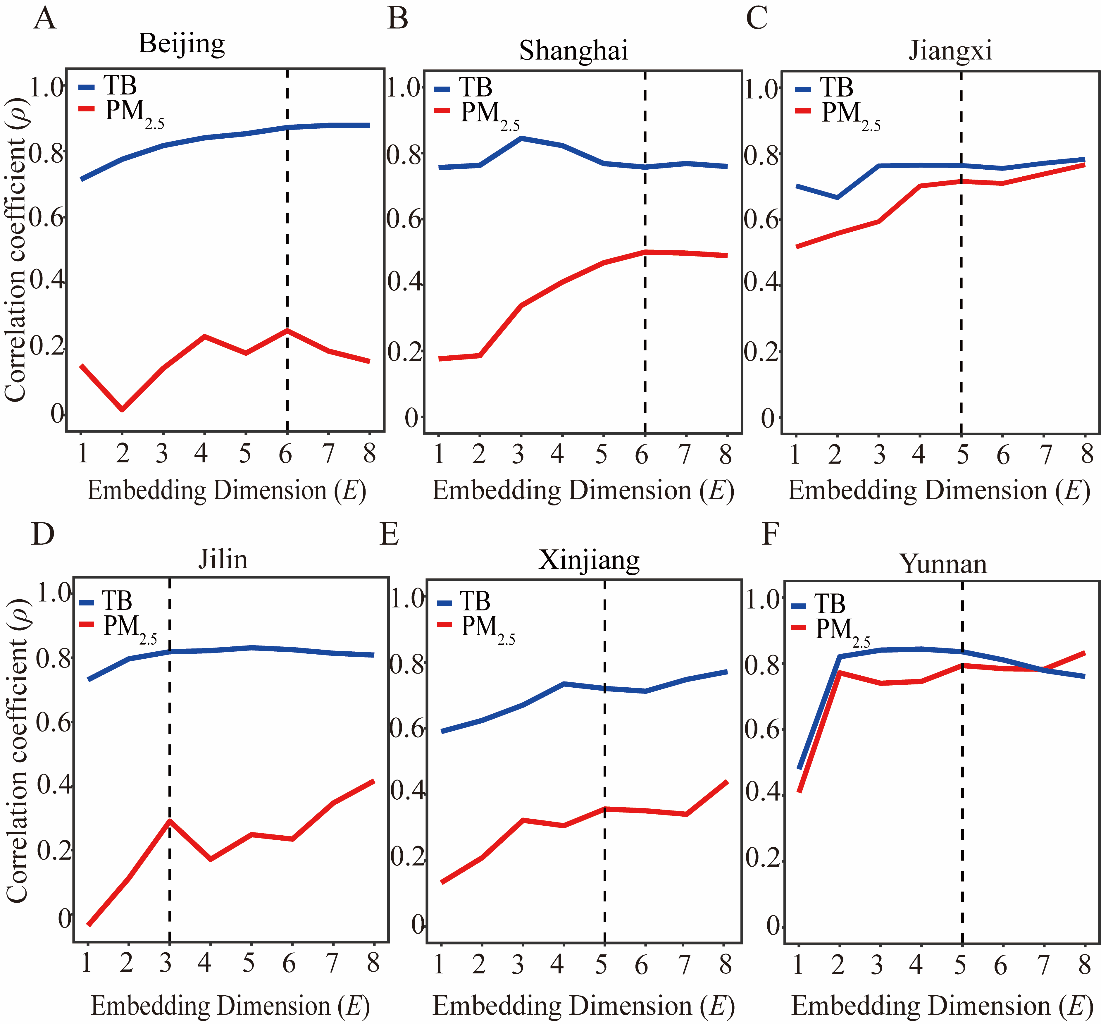


**Figure S8**. Optimal embedding dimension for six representative provinces in China.

For each univariate SSR, the correlation coefficient 𝜌 for TB (blue lines) and PM_2.5_ (red lines) are plotted against different values of embedding dimension in (A) Beijing, (B) Shanghai, (C) Jiangxi, (D) Jilin, (E) Xinjiang and (F) Yunan. The optimal dimension corresponds to the most accurate prediction (peaks).

**Figure S9**


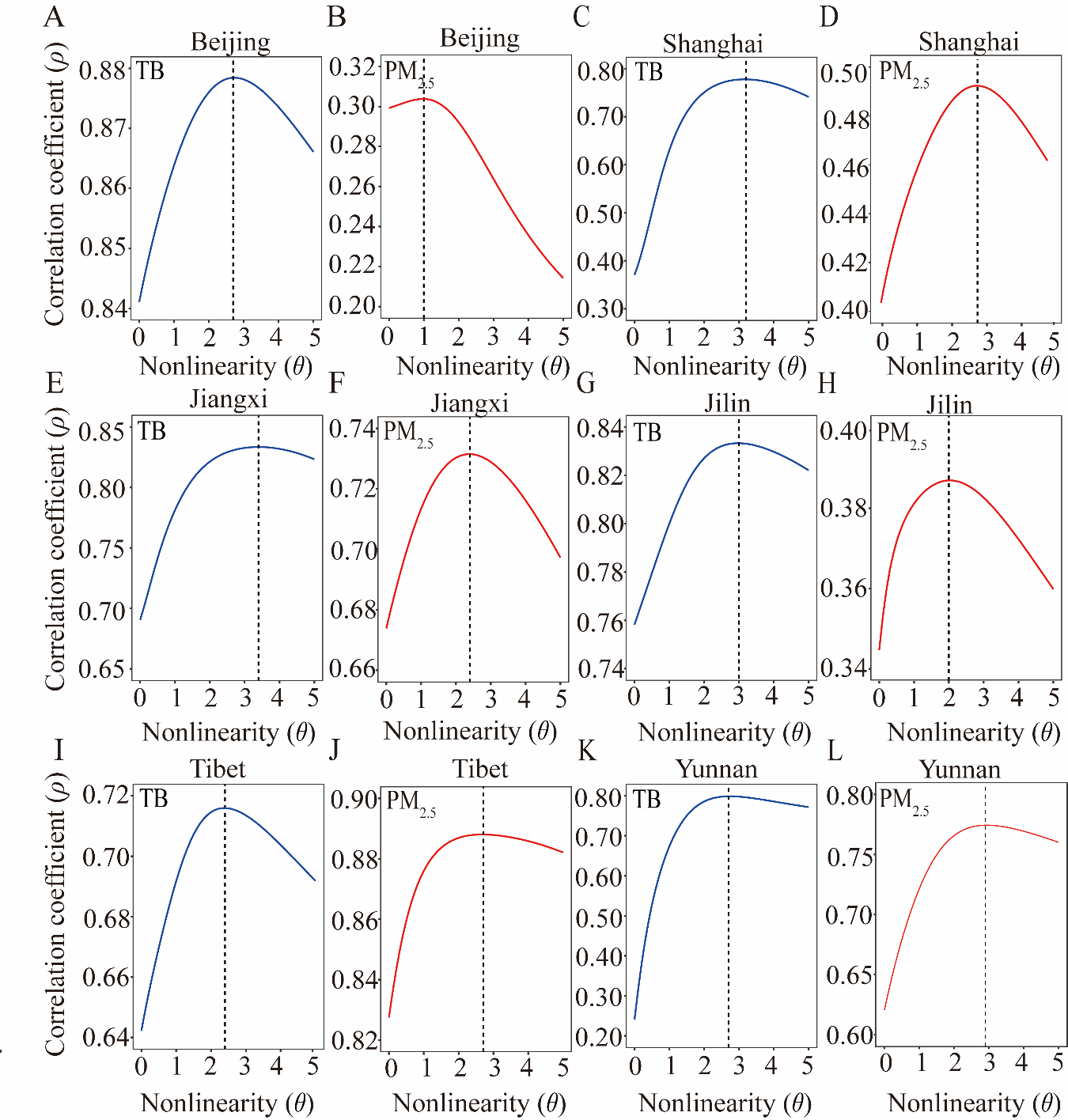


**Figure S9**. Nonlinearity test results for six representative provinces in China.

For each univariate SSR, the nonlinearity coefficient θ for TB and PM_2.5_ are plotted to prediction in (A, B) Beijing, (C, D) Shanghai, (E, F) Jiangxi, (G, H) Jilin, (I, J) Xinjiang and (K, L) Yunan. Optimal 𝜃 value > 0 indicates the nonlinear dynamic system.

**Figure S10**


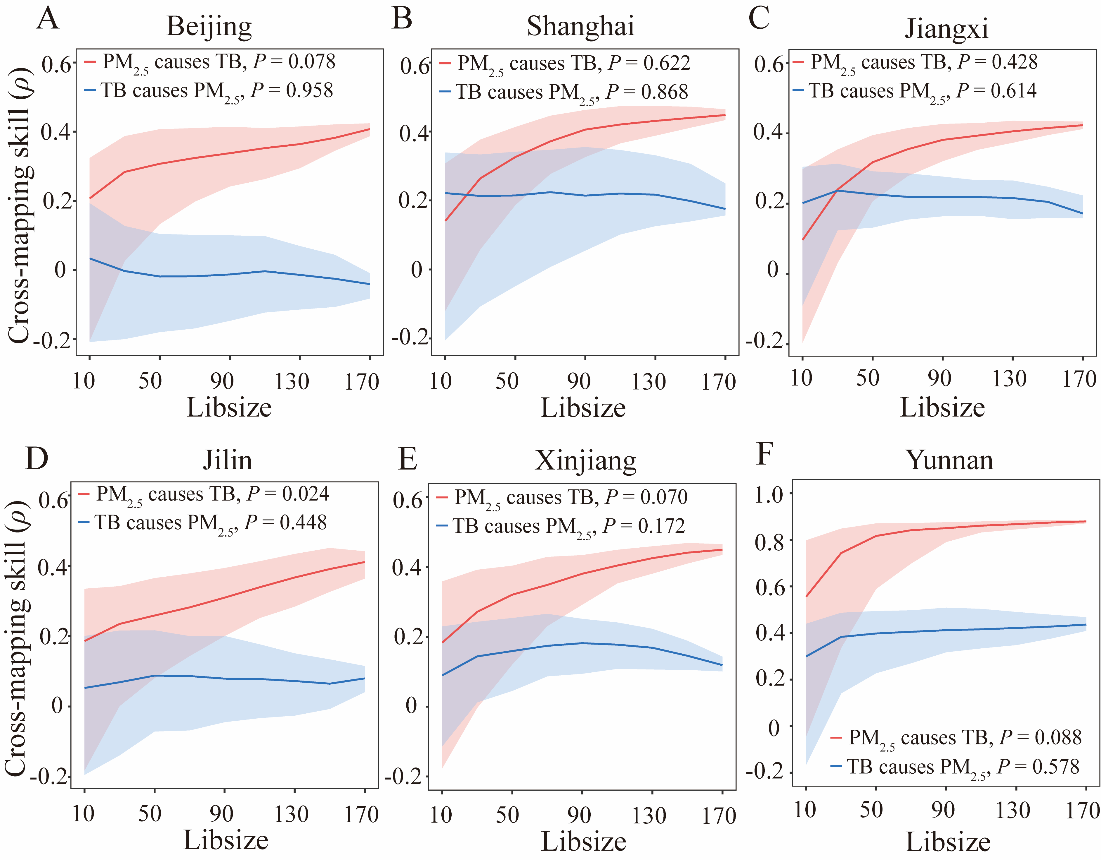


**Figure S10**. Causality between PM_2.5_ and TB incidence for six representative provinces in China.

For cross-mapping between PM_2.5_ and TB incidence, the increase of time series shows significant convergence in cross-mapping skill (ρ), indicating putative causality. The solid lines show the medians of the predictive power, and the shaded regions represent the median plus or minus its 0.025 and 0.975 quartiles of the bootstrapped time-series segments obtained from 500 runs of randomized time series with conserved seasonal trends. The causality is considered significant if *P* ≤ 0.1.

**Table S1.** Summary statistics of average annual TB incidence, PM_2.5_ concentration and socio-economic covariates in China during 1982-2019

| **Variable** | **Mean** | **SD** | **Min** | **P_25_** | **P_50_** | **P_75_** | **Max** |
| --- | --- | --- | --- | --- | --- | --- | --- |
| TB incidence (per 10^5^ population) | 44.35 | 19.06 | 9.78 | 28.85 | 39.69 | 61.74 | 74.32 |
| PM_2.5_ (μg/m^3^) | 28.31 | 5.24 | 20.48 | 23.36 | 27.51 | 34.19 | 35.94 |
| Birth rate (‰) | 15.62 | 4.05 | 10.48 | 12.10 | 13.71 | 19.74 | 23.33 |
| Population density ( per km^2^) | 129.97 | 12.23 | 105.92 | 120.30 | 132.53 | 139.94 | 146.93 |
| Real per capita GDP (constant 2010 USD) | 3877.06 | 3289.95 | 533 | 1062.41 | 2525.30 | 6409.49 | 11285.82 |
| Certified doctors (per 10^4^ population) | 17.24 | 3.47 | 12.9 | 15.75 | 16 | 18 | 28 |
| Beds of medical institutions (per 10^4^ population) | 30.83 | 12.47 | 20.21 | 23.2 | 23.95 | 36.45 | 63 |
|  | **Time period** | | **Begin** | **End** | **Average** | **Annual rate of increase** | |
| TB incidence | 1982-2001 | | 9.78 | 36.97 | 28.91 | 7.25% | |
|  | 2001-2007 | | 36.97 | 74.32 | 56.85 | 12.34% | |
|  | 2007-2019 | | 74.32 | 51.73 | 63.13 | -2.97% | |
| PM_2.5_ | 1982-1995 | | 20.48 | 23.96 | 22.90 | 1.21% | |
|  | 1995-2008 | | 23.96 | 35.94 | 29.52 | 3.17% | |
|  | 2008-2019 | | 35.94 | 31.16 | 33.49 | -1.29% | |

**Table S2.** Stationarity, unit root and cointegration tests for the PM_2.5_ and tuberculosis series

| **Test** | **Null hypothesis (H_0_)** | **Lag** | **Statistic** | ***P*** | **Conclusion** |
| --- | --- | --- | --- | --- | --- |
| KPSS (no trend) | TB is level statationary | 2 | 1.110 | ≤0.01 | Non-stationary |
|  | PM_2.5_ is level statationary | 2 | 1.130 | ≤0.01 | Non-stationary |
| DF-GLS (no trend) | TB has unit root | 2 | -0.760 | >0.1 | Unit root |
|  | PM_2.5_ has unit root | 2 | -0.437 | >0.1 | Unit root |
| KPSS (no trend) | ΔTB is level statationary | 1 | 0.290 | >0.1 | Stationary |
|  | ΔPM_2.5_ is level statationary | 1 | 0.208 | >0.1 | Stationary |
| DF-GLS (no trend) | ΔTB has unit root | 1 | -2.669 | ≤0.01 | No unit root |
|  | ΔPM_2.5_ has unit root | 1 | -3.726 | ≤0.01 | No unit root |
| Johansen | rank ≤ 0 |  | 15.6883 | ≤0.05 | Co-integrated at rank = 1 |
|  | rank ≤ 1 |  | 2.4275 | >0.05 |  |

KPSS, Kwiatkowski, Phillips, Schmidt and Shin test; DF-GLS, augmented Dickey-Fuller test based on GLS-detrending; Δ, first difference

**Table S3.** Summary statistics of average annual TB incidence and PM_2.5_ concentration in 31 provinces of China during 1997-2018.

| Province | Mean | SD | Min | P_25_ | P_50_ | P_75_ | Max |
| --- | --- | --- | --- | --- | --- | --- | --- |
| TB incidence (per 10^5^ population) | | | | | | | |
| Heilongjiang | 82.06 | 21.65 | 48.57 | 59.93 | 87.48 | 101.42 | 126.47 |
| Inner Mongolia | 60.12 | 21.99 | 25.08 | 45.15 | 51.68 | 78.77 | 108.22 |
| Jilin | 63.90 | 18.27 | 36.29 | 49.18 | 61.35 | 81.22 | 100.08 |
| Liaoning | 50.04 | 13.42 | 23.38 | 36.18 | 55.23 | 59.12 | 70.52 |
| Xinjiang | 152.98 | 65.43 | 12.45 | 88.69 | 174.36 | 194.44 | 304.94 |
| Beijing | 34.65 | 14.32 | 16.03 | 17.77 | 33.56 | 47.62 | 58.64 |
| Hebei | 45.80 | 14.23 | 20.98 | 33.32 | 47.13 | 59.56 | 65.57 |
| Tianjin | 31.36 | 8.80 | 19.52 | 22.05 | 32.63 | 37.80 | 50.15 |
| Henan | 61.78 | 25.72 | 17.53 | 37.33 | 64.62 | 75.46 | 115.17 |
| Shanxi | 45.92 | 23.16 | 13.68 | 21.91 | 44.33 | 69.92 | 88.32 |
| Ningxia | 47.54 | 14.54 | 22.61 | 36.40 | 45.29 | 57.92 | 76.86 |
| Shandong | 32.52 | 9.78 | 17.60 | 21.44 | 33.24 | 42.30 | 46.78 |
| Gansu | 73.85 | 24.84 | 38.71 | 56.14 | 66.27 | 90.04 | 128.29 |
| Qinghai | 84.51 | 32.18 | 35.82 | 48.10 | 88.39 | 107.25 | 140.33 |
| Shaanxi | 59.16 | 24.00 | 19.16 | 36.62 | 58.19 | 77.90 | 107.48 |
| Jiangsu | 47.63 | 12.30 | 31.02 | 37.88 | 43.65 | 58.83 | 77.00 |
| Anhui | 56.75 | 21.33 | 23.03 | 34.45 | 57.61 | 66.63 | 97.86 |
| Tibet | 97.39 | 44.23 | 26.03 | 51.70 | 96.20 | 138.64 | 166.66 |
| Shanghai | 38.10 | 11.42 | 26.14 | 29.13 | 34.27 | 50.91 | 60.46 |
| Hubei | 77.76 | 23.04 | 43.55 | 53.48 | 78.86 | 92.89 | 120.17 |
| Sichuan | 72.45 | 18.59 | 48.10 | 56.32 | 68.40 | 85.47 | 112.00 |
| Chongqing | 90.51 | 25.81 | 48.10 | 74.59 | 84.44 | 105.54 | 151.51 |
| Zhejiang | 61.71 | 15.82 | 45.26 | 49.94 | 53.96 | 73.62 | 92.90 |
| Jiangxi | 72.62 | 23.25 | 32.28 | 51.77 | 72.00 | 88.26 | 134.52 |
| Hunan | 73.05 | 23.59 | 36.36 | 41.24 | 80.87 | 90.97 | 104.15 |
| Guizhou | 120.37 | 34.15 | 62.63 | 91.45 | 129.27 | 137.26 | 182.99 |
| Fujian | 58.03 | 18.56 | 30.89 | 45.13 | 52.15 | 71.93 | 98.11 |
| Yunnan | 51.43 | 12.22 | 30.12 | 37.10 | 55.81 | 59.59 | 73.70 |
| Guangxi | 93.98 | 26.23 | 42.88 | 73.19 | 96.70 | 106.76 | 145.67 |
| Guangdong | 72.21 | 23.08 | 38.68 | 47.05 | 74.39 | 95.44 | 109.70 |
| Hainan | 91.00 | 35.06 | 27.93 | 41.81 | 96.14 | 113.40 | 143.62 |
| PM_2.5_ (μg/m^3^) | | | | | | | |
| Heilongjiang | 18.61 | 4.53 | 12.49 | 14.76 | 18.94 | 21.68 | 29.65 |
| Inner Mongolia | 19.59 | 2.53 | 16.42 | 17.80 | 19.35 | 20.66 | 28.04 |
| Jilin | 21.63 | 3.69 | 15.20 | 19.08 | 21.82 | 23.91 | 30.91 |
| Liaoning | 29.75 | 4.81 | 20.66 | 25.11 | 30.77 | 33.21 | 38.69 |
| Xinjiang | 34.55 | 3.40 | 28.06 | 32.59 | 34.07 | 36.28 | 42.51 |
| Beijing | 43.70 | 6.20 | 31.08 | 37.78 | 45.09 | 49.19 | 50.97 |
| Hebei | 44.03 | 6.36 | 31.14 | 38.99 | 47.00 | 49.19 | 50.65 |
| Tianjin | 47.54 | 7.89 | 31.74 | 41.29 | 50.90 | 53.94 | 56.04 |
| Henan | 52.99 | 7.20 | 38.26 | 48.49 | 54.79 | 58.97 | 60.87 |
| Shanxi | 34.33 | 3.53 | 27.68 | 32.14 | 34.51 | 37.96 | 38.80 |
| Ningxia | 32.74 | 2.48 | 28.40 | 31.06 | 32.56 | 34.49 | 38.32 |
| Shandong | 43.20 | 7.33 | 28.64 | 37.95 | 46.92 | 49.05 | 50.66 |
| Gansu | 25.72 | 1.94 | 22.56 | 24.02 | 25.81 | 27.45 | 28.64 |
| Qinghai | 12.12 | 1.13 | 9.82 | 11.39 | 12.41 | 13.11 | 13.63 |
| Shaanxi | 34.92 | 3.48 | 28.47 | 32.74 | 34.48 | 38.44 | 40.50 |
| Jiangsu | 43.70 | 6.85 | 29.64 | 38.96 | 46.15 | 48.96 | 51.27 |
| Anhui | 44.59 | 6.97 | 31.06 | 39.67 | 47.60 | 50.10 | 52.32 |
| Tibet | 5.14 | 0.70 | 3.23 | 4.91 | 5.18 | 5.38 | 6.66 |
| Shanghai | 36.30 | 5.54 | 23.78 | 32.27 | 37.76 | 40.55 | 43.07 |
| Hubei | 45.33 | 6.44 | 32.58 | 41.40 | 46.52 | 51.18 | 52.27 |
| Sichuan | 39.24 | 4.23 | 31.50 | 36.90 | 38.93 | 44.04 | 45.59 |
| Chongqing | 44.83 | 5.18 | 34.50 | 41.54 | 44.36 | 49.27 | 52.56 |
| Zhejiang | 33.51 | 4.52 | 23.98 | 30.46 | 35.21 | 37.02 | 39.17 |
| Jiangxi | 37.92 | 5.78 | 37.13 | 33.55 | 39.27 | 43.27 | 45.14 |
| Hunan | 43.11 | 6.37 | 31.30 | 39.31 | 43.92 | 49.11 | 50.93 |
| Guizhou | 33.41 | 4.40 | 26.09 | 29.88 | 33.14 | 37.52 | 39.91 |
| Fujian | 31.11 | 3.75 | 23.92 | 28.13 | 31.86 | 34.13 | 36.00 |
| Yunnan | 18.33 | 2.16 | 14.54 | 16.44 | 18.50 | 19.94 | 22.28 |
| Guangxi | 33.38 | 5.22 | 24.15 | 28.56 | 34.41 | 37.71 | 40.49 |
| Guangdong | 31.62 | 4.77 | 22.86 | 27.12 | 32.54 | 35.70 | 38.09 |
| Hainan | 21.39 | 3.04 | 14.55 | 19.07 | 21.85 | 23.82 | 25.40 |

The provinces were ordered by their central latitudes.

**Table S4**. Panel unit root and cross-sectional dependence tests for the annual data covering 1997-2018.

| **Test** | H_0_ | Statistic | *P* | Conclusion |
| --- | --- | --- | --- | --- |
| LLC (intercept) | TB panels contains unit roots | -4.5618 | ≤0.0001 | No unit root/Stationary |
|  | PM_2.5_ panels contains unit roots | -2.0693 | 0.0193 | No unit root/Stationary |
| LLC (intercept and trend) | TB panels contains unit roots | -1.9075 | 0.0282 | No unit root/Stationary |
|  | PM_2.5_ panels contains unit roots | 2.7640 | 0.9971 | Unit root/Non-stationary |
| LLC (none) | TB panels contains unit roots | -1.0684 | 0.1427 | Unit root/Non-stationary |
|  | PM_2.5_ panels contains unit roots | -3.1871 | 0.0007 | No unit root/Stationary |
| Frees test | Panels are cross-sectional independent | 8.528 | ≤0.01 | Cross-sectional dependence |

LLC, Levin-Lin-Chu unit-root test.

**Table S5**. Summary statistics of monthly TB incidence and average PM_2.5_ concentration in 31 provinces of China during 2004-2018

| Province | Mean | SD | Min | P_25_ | P_50_ | P_75_ | Max |
| --- | --- | --- | --- | --- | --- | --- | --- |
| TB incidence (per 10^5^ population) | | | | | | | |
| Heilongjiang | 93.65 | 15.27 | 65.23 | 86.73 | 89.43 | 102.93 | 126.47 |
| Inner Mongolia | 70.07 | 19.07 | 47.71 | 49.19 | 67.39 | 88.11 | 108.22 |
| Jilin | 71.20 | 16.09 | 47.43 | 56.45 | 72.57 | 83.79 | 100.08 |
| Liaoning | 58.28 | 5.28 | 51.40 | 55.16 | 56.95 | 60.10 | 70.52 |
| Xinjiang | 190.55 | 36.32 | 142.01 | 172.73 | 185.66 | 202.59 | 304.94 |
| Beijing | 42.61 | 9.68 | 30.43 | 32.74 | 41.29 | 51.29 | 58.64 |
| Hebei | 53.85 | 8.53 | 39.31 | 46.11 | 53.92 | 62.54 | 65.57 |
| Tianjin | 27.04 | 6.43 | 19.52 | 21.27 | 25.54 | 33.98 | 37.94 |
| Henan | 75.77 | 17.13 | 55.16 | 62.74 | 69.91 | 92.85 | 115.17 |
| Shanxi | 58.45 | 16.16 | 35.03 | 42.32 | 58.41 | 71.06 | 88.32 |
| Ningxia | 53.62 | 12.12 | 36.73 | 43.00 | 50.84 | 66.08 | 76.86 |
| Shandong | 38.05 | 6.12 | 28.12 | 33.06 | 38.16 | 43.73 | 46.78 |
| Gansu | 81.96 | 26.20 | 38.71 | 58.13 | 76.26 | 108.23 | 128.29 |
| Qinghai | 102.50 | 21.06 | 71.78 | 88.37 | 95.30 | 123.26 | 140.33 |
| Shaanxi | 71.96 | 16.04 | 55.90 | 57.39 | 67.81 | 87.12 | 107.48 |
| Jiangsu | 50.99 | 13.57 | 31.02 | 39.52 | 48.69 | 62.97 | 77.00 |
| Anhui | 68.33 | 14.75 | 51.08 | 56.77 | 62.90 | 85.63 | 97.86 |
| Tibet | 121.38 | 30.80 | 66.55 | 91.17 | 123.03 | 148.00 | 166.66 |
| Shanghai | 32.67 | 6.47 | 26.14 | 27.56 | 30.38 | 35.69 | 50.70 |
| Hubei | 89.76 | 16.65 | 63.49 | 78.14 | 87.67 | 107.55 | 120.17 |
| Sichuan | 81.22 | 15.85 | 57.34 | 67.13 | 78.84 | 95.77 | 112.00 |
| Chongqing | 98.43 | 25.65 | 73.34 | 76.62 | 88.82 | 125.16 | 151.51 |
| Zhejiang | 66.18 | 17.39 | 45.26 | 50.80 | 59.56 | 86.27 | 92.90 |
| Jiangxi | 84.80 | 16.96 | 70.83 | 71.99 | 79.68 | 94.35 | 134.52 |
| Hunan | 87.41 | 10.95 | 64.20 | 78.75 | 88.59 | 92.87 | 104.14 |
| Guizhou | 139.75 | 19.43 | 114.06 | 129.14 | 133.46 | 157.35 | 182.99 |
| Fujian | 62.38 | 19.54 | 42.74 | 45..24 | 52.58 | 83.74 | 98.11 |
| Yunnan | 58.97 | 4.85 | 53.67 | 55.60 | 58.01 | 60.42 | 73.70 |
| Guangxi | 106.96 | 18.96 | 82.82 | 96.41 | 100.65 | 127.23 | 145.67 |
| Guangdong | 85.38 | 14.45 | 64.81 | 74.12 | 78.07 | 99.51 | 109.70 |
| Hainan | 110.94 | 21.18 | 84.18 | 95.27 | 107.07 | 138.31 | 143.63 |
| PM_2.5_ (μg/m^3^) | | | | | | | |
| Heilongjiang | 19.22 | 3.56 | 12.93 | 16.46 | 19.31 | 21.62 | 25.45 |
| Inner Mongolia | 19.61 | 1.76 | 16.56 | 18.21 | 20.10 | 20.70 | 22.62 |
| Jilin | 22.67 | 2.24 | 19.56 | 21.13 | 22.34 | 24.34 | 26.83 |
| Liaoning | 31.60 | 2.88 | 25.84 | 29.52 | 31.69 | 34.09 | 37.10 |
| Xinjiang | 35.88 | 3.03 | 29.43 | 33.96 | 36.19 | 36.47 | 42.51 |
| Beijing | 46.84 | 3.29 | 41.52 | 43.33 | 47.70 | 49.70 | 50.97 |
| Hebei | 47.32 | 3.04 | 41.04 | 43.86 | 48.75 | 49.24 | 50.65 |
| Tianjin | 51.59 | 3.70 | 43.13 | 49.07 | 52.54 | 54.14 | 56.04 |
| Henan | 56.80 | 3.47 | 50.46 | 53.06 | 58.21 | 59.35 | 60.87 |
| Shanxi | 35.87 | 2.58 | 32.17 | 32.53 | 36.59 | 38.07 | 38.80 |
| Ningxia | 33.38 | 2.55 | 29.32 | 31.70 | 33.57 | 35.25 | 38.32 |
| Shandong | 47.08 | 3.29 | 38.82 | 44.64 | 48.28 | 49.27 | 50.66 |
| Gansu | 26.39 | 1.73 | 23.94 | 24.59 | 26.99 | 27.79 | 28.64 |
| Qinghai | 12.49 | 0.90 | 10.86 | 11.64 | 12.88 | 13.18 | 13.63 |
| Shaanxi | 36.40 | 2.78 | 32.36 | 33.43 | 36.66 | 38.84 | 40.50 |
| Jiangsu | 47.25 | 3.09 | 39.89 | 45.28 | 48.36 | 49.38 | 51.27 |
| Anhui | 48.09 | 3.65 | 40.28 | 44.99 | 48.91 | 51.23 | 52.32 |
| Tibet | 5.37 | 0.51 | 4.74 | 4.95 | 5.26 | 5.48 | 6.66 |
| Shanghai | 39.02 | 2.69 | 32.74 | 37.57 | 39.84 | 41.11 | 43.07 |
| Hubei | 48.60 | 3.74 | 41.51 | 45.95 | 50.36 | 51.70 | 52.27 |
| Sichuan | 41.36 | 2.95 | 37.12 | 38.22 | 41.25 | 44.14 | 45.59 |
| Chongqing | 47.54 | 3.34 | 42.82 | 43.53 | 47.46 | 50.40 | 52.56 |
| Zhejiang | 35.77 | 2.50 | 30.90 | 33.88 | 35.99 | 37.81 | 39.17 |
| Jiangxi | 40.78 | 3.66 | 33.65 | 38.67 | 42.49 | 43.41 | 45.14 |
| Hunan | 46.43 | 3.91 | 39.56 | 43.56 | 48.45 | 49.34 | 50.93 |
| Guizhou | 35.74 | 3.02 | 30.75 | 32.62 | 36.05 | 38.10 | 39.91 |
| Fujian | 33.10 | 2.31 | 28.95 | 31.26 | 33.63 | 35.25 | 36.00 |
| Yunnan | 19.41 | 1.55 | 17.32 | 18.29 | 18.89 | 20.31 | 22.28 |
| Guangxi | 36.22 | 3.04 | 31.08 | 33.66 | 37.52 | 37.91 | 40.49 |
| Guangdong | 34.25 | 2.59 | 29.98 | 31.58 | 35.01 | 36.21 | 38.09 |
| Hainan | 23.04 | 1.59 | 20.04 | 21.83 | 23.56 | 24.14 | 25.40 |

The provinces were ordered by their central latitudes.

**Table S6**. Panel unit root and cross-sectional dependence tests for the monthly data covering 2004-2018.

| **Test** | **H_0_** | **Statistic** | ***P*** | **Conclusion** |
| --- | --- | --- | --- | --- |
| LLC (intercept) | TB panels contain unit roots | 2.408 | 0.992 | unit root/Non-stationary |
|  | PM_2.5_ panels contain unit roots | -9.762 | ≤0.001 | No unit root/Stationary |
| LLC (intercept and trend) | TB panels contain unit roots | 3.842 | 1.000 | unit root/Non-stationary |
|  | PM_2.5_ panels contain unit roots | -11.1262 | ≤0.001 | No unit root/Stationary |
| LLC (none) | TB panels contain unit roots | -4.535 | ≤0.001 | No unit root/Stationary |
|  | PM_2.5_ panels contain unit roots | -7.466 | ≤0.001 | No unit root/Stationary |
| Frees | Panels are cross-sectional independent | 15.943 | ≤0.001 | cross-sectional independence |

LLC, Levin-Lin-Chu unit-root test.

**Extended References**

1. Schwoebel V: **Surveillance of tuberculosis**. *Indian J Tuberc* 2020, **67**(4S):S33-S42.

2. Liang S, Yang C, Zhong B, Guo J, Li H, Carlton EJ, Freeman MC, Remais JV: **Surveillance systems for neglected tropical diseases: global lessons from China's evolving schistosomiasis reporting systems, 1949-2014**. *Emerg Themes Epidemiol* 2014, **11**:19.

3. Gelaro R, McCarty W, Suárez MJ, Todling R, Molod A, Takacs L, Randles C, Darmenov A, Bosilovich MG, Reichle R *et al*: **The Modern-Era Retrospective Analysis for Research and Applications, Version 2 (MERRA-2)**. *J Clim* 2017, **Volume 30**(Iss 13):5419-5454.

4. Ma J, Xu J, Qu Y: **Evaluation on the surface PM2.5 concentration over China mainland from NASA's MERRA-2**. *Atmospheric Environment* 2020, **237**:117666.

5. Buchard V, da Silva AM, Randles CA, Colarco P, Ferrare R, Hair J, Hostetler C, Tackett J, Winker D: **Evaluation of the surface PM2.5 in Version 1 of the NASA MERRA Aerosol Reanalysis over the United States**. *Atmospheric Environment* 2016, **125**:100-111.

6. Rodrigues SD, Ueda RM, Barreto AC, Zanini RR, Souza AM: **How atmospheric pollutants impact the development of chronic obstructive pulmonary disease and lung cancer: A var-based model**. *Environ Pollut* 2021, **275**:116622.

7. Alimi S: **Export - Led Growth or Growth – Driven Exports? Evidence from Nigeria**. *British Journal of Economics, Management & Trade* 2013, **3**(2):89-100.

8. Holtz-Eakin D, Newey W, Rosen HS: **Estimating Vector Autoregressions with Panel Data**. *Econometrica* 1988, **56**(6):1371-1395.

9. Dumitrescu E-I, Hurlin C: **Testing for Granger non-causality in heterogeneous panels**. *Economic Modelling* 2012, **29**(4):1450-1460.

10. Germano-Soares AH, Andrade-Lima A, Meneses AL, Correia MA, Parmenter BJ, Tassitano RM, Cucato GG, Ritti-Dias RM: **Association of time spent in physical activities and sedentary behaviors with carotid-femoral pulse wave velocity: A systematic review and meta-analysis**. *Atherosclerosis* 2018, **269**:211-218.

11. Sugihara G, May R, Ye H, Hsieh CH, Deyle E, Fogarty M, Munch S: **Detecting causality in complex ecosystems**. *Science* 2012, **338**(6106):496-500.

12. Schiecke K, Pester B, Feucht M, Leistritz L, Witte H: **Convergent Cross Mapping: Basic concept, influence of estimation parameters and practical application**. *Annu Int Conf IEEE Eng Med Biol Soc* 2015, **2015**:7418-7421.

13. Deyle ER, Maher MC, Hernandez RD, Basu S, Sugihara G: **Global environmental drivers of influenza**. *Proc Natl Acad Sci U S A* 2016, **113**(46):13081-13086.

14. Nova N, Deyle ER, Shocket MS, MacDonald AJ, Childs ML, Rypdal M, Sugihara G, Mordecai EA: **Susceptible host availability modulates climate effects on dengue dynamics**. *Ecol Lett* 2021, **24**(3):415-425.

15. Sugihara G, May RM: **Nonlinear forecasting as a way of distinguishing chaos from measurement error in time series**. *Nature* 1990, **344**(6268):734-741.

16. Huang K, Ding K, Yang XJ, Hu CY, Jiang W, Hua XG, Liu J, Cao JY, Zhang T, Kan XH *et al*: **Association between short-term exposure to ambient air pollutants and the risk of tuberculosis outpatient visits: A time-series study in Hefei, China**. *Environ Res* 2020, **184**:109343.
